# Nasal pathobiont abundance does not differ between dairy cattle with or without clinical symptoms of bovine respiratory disease

**DOI:** 10.1186/s42523-025-00382-3

**Published:** 2025-02-18

**Authors:** Ruth Eunice Centeno-Delphia, Erica A. Long, Audrey C. Ellis, Sarah Hofmann, Kara Mosier, Noelmi Ulloa, Johnnie Junior Cheng, Andrew Richards, Jacquelyn P. Boerman, Jennifer Koziol, Mohit S. Verma, Timothy A. Johnson

**Affiliations:** 1 Department of Animal Science, Purdue University, 270 S Russell St, West Lafayette, IN USA; 2 Escuela Agrícola Panamericana Zamorano, Valle del Yeguare, Honduras; 3 Department of Agricultural and Biological Engineering, Purdue University, West Lafayette, IN USA; 4 School of Veterinary Medicine, Texas Tech University, Amarillo, TX USA; 5 Weldon School of Biomedical Engineering, Purdue University, West Lafayette, IN USA; 6 Brick Nanotechnology Center, Purdue University, West Lafayette, IN USA

**Keywords:** Dairy calves, 16S rRNA gene, qPCR, BRD-pathobionts, Bovine respiratory disease

## Abstract

**Background:**

Bovine respiratory disease (BRD) remains a significant health and economic problem to the dairy cattle industry. Multiple risk factors contribute to BRD susceptibility including the bacterial pathobionts *Mannheimia haemolytica*, *Pasteurella multocida*, *Histophilus somni*, and *Mycoplasma bovis*. Studies have characterized and quantified the abundance of these bacteria in the nasal cavity of cattle to infer and help disease diagnosis; nonetheless, there is still discrepancy in the results observed of when these microbes are commensal or pathogenic. Additionally, some of these studies are limited to a specific farm. The goal of this study is to compare the nasal microbiome community (diversity and composition) and the abundance of the four bacterial pathogens (by qPCR) in the nasal cavity to identify differences between dairy calves that are apparently healthy and those identified to have BRD. Nasal swabs were collected from approximately 50 apparently healthy and 50 BRD-affected calves sampled from five different dairy farms in the US (CA, IN, NY (two farms), and TX).

**Results:**

Calves diagnosed with BRD in NY, and TX had lower nasal microbiome diversity compared to the apparently healthy calves. Differences in the nasal microbiome composition were observed between the different farms predicted by Bray-Curtis and weighted UniFrac dissimilarities. Commensal and pathobiont genera *Acinetobacter*, *Moraxella*, *Psychrobacter*, *Histophilus*, *Mannheimia*, *Mycoplasma*, and *Pasteurella* were prevalent in the bovine nasal microbiome regardless of farm or disease status. The BRD-pathobiont *H. somni* was the most prevalent pathobiont among all the samples and *M. bovis* the least prevalent. Only in CA was the abundance of a pathobiont different according to disease status, where *M. haemolytica* was significantly more abundant in the BRD-affected animals than apparently healthy animals.

**Conclusions:**

This study offers insight into the nasal microbiome community composition in both animals diagnosed with BRD and healthy animals, and shows that the farm effect plays a more significant role in determining the microbiome community than disease status in young dairy calves.

**Supplementary Information:**

The online version contains supplementary material available at 10.1186/s42523-025-00382-3.

## Background

Development of bovine respiratory disease (BRD) involves multifactorial interactions, including predisposing, environmental, and epidemiological factors [1, 2]. The combination of these multiple factors typically leads to bacterial and/or viral respiratory tract infections in cattle, resulting in economic losses due to increased morbidity, mortality, treatment costs, and reduced production [2–4]. In the dairy cattle industry, including calves and cows, respiratory disease accounts for the highest percentage of cattle deaths (16%) [5]. Young calves, particularly during the first weeks of life, are more susceptible to BRD due to their naive immune systems [6]. Currently, diagnosing BRD relies on observing clinical signs such as changes in feed intake, depression, alterations in respiratory function and elevated rectal temperature [7]. However, based on a meta-analysis, detection of BRD clinical signs has low sensitivity (62%) and specificity (63%) in correctly diagnosing the animals [8]. Studies have indicated that the composition of the respiratory microbiome can serve as an indicator of disease status [9–14]. Specifically, the bacteria *H. somni*, *P. multocida*, *M. haemolytica*, and *M. bovis* have been associated with BRD cases [9, 15–17]. However, since these microbes are common among both healthy and BRD-affected animals, they can also be referred to as pathobionts. When the animal immune system is weakened, they can proliferate in the upper respiratory tract and invade the lungs, causing infection [18]. Further investigation has identified specific serotypes (*M. haemolytica* serotypes A1 and A6, *P. multocida*, serotype A3) or strains (*H. somni*, strain 2336) related to BRD mortalities [19–24].

With advancements in sequencing technology, it has become possible to identify the cattle commensal respiratory microbial community and its role in animal health. Nevertheless, as more knowledge is acquired, studies have identified geography/farm as a driving factor influencing the respiratory microbiome [25]. For example, a study performed by Karle et al., (2019) [26] compared the BRD incidence in three distinct dairy regions in California: Northern California (9.30% incidence), Northern San Joaquin Valley (4.51%), and Greater Southern California (7.35%). Additionally, the authors identified that within-farm management practices, including colostrum management, group housing, and feeding salable milk, were associated with BRD incidence. Therefore, the current observational study aims to characterize the nasal microbiome, sampled across different farms in the U.S. and identify the abundance of BRD-associated pathobionts in animals diagnosed with BRD compared to the healthy group. Nasal swabs were collected from five dairy farms across the United States (one farm located in IN, CA, and TX and two farms in NY) to account for the effect of farm and find potential microbiome traits that are common among the farm as indicators of disease status. We hypothesized that the nasal microbiome in animals identified with BRD would exhibit a decrease in alpha diversity, and an altered community composition, including an increase in the abundance of BRD pathobionts as compared to the healthy animals.

## Results

### Dairy calf nasal microbiome taxonomical composition

**Fig. 1:**
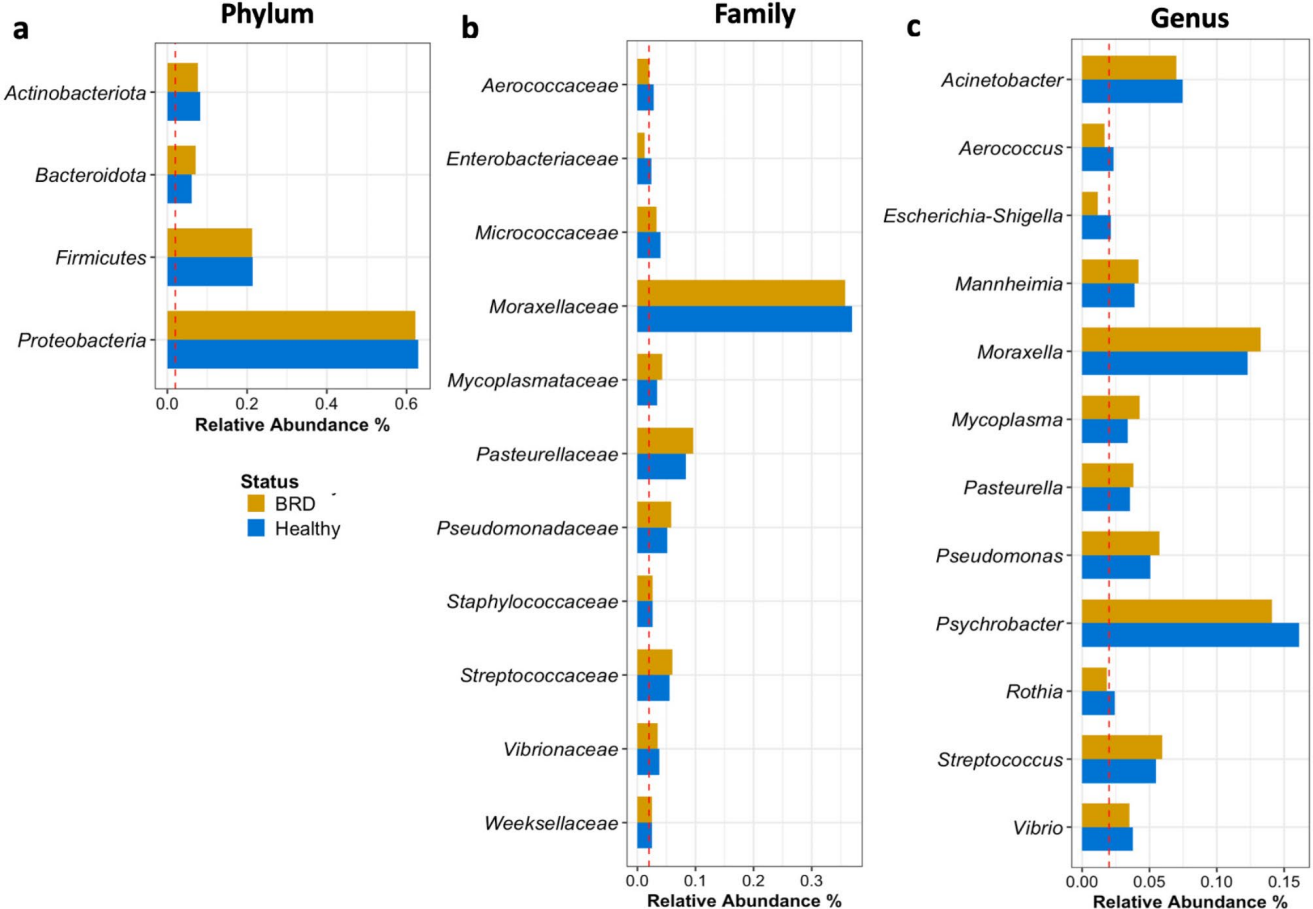
Dairy cattle nasal microbiome taxa with an average relative abundance > 2% per sample at the phylum (**a**), family (**b**) and genus (**c**) taxonomic levels in BRD-affected and apparently healthy animals. If only one group (apparently healthy or BRD) surpassed the 2% threshold (red dashed line), then both groups were reported

In this study, the most prevalent taxonomic groups (average relative abundance > 0.02 or 2% per sample) in both study groups varied by farm and health status. The predominant phyla in the nasal microbiome of dairy calves were *Proteobacteria* (60% of the community on average), *Firmicutes* (21%) regardless of disease status and farm, followed by *Actinobacteriota* and *Bacteroides* (8%) (Fig. 1a). At the family level, *Moraxellaceae* (36%) and *Pasteurellaceae* (9%) were the most abundant in the nasal cavity (Fig. 1b). At the genus level, the groups *Psychrobacter*, *Moraxella*, and *Acinetobacter* were the most abundant in the nasal cavity. The genera *Mannheimia*, *Mycoplasma*, and *Pasteurella* were identified as prominent members of the nasal microbiome (even in healthy animals), with a slight numerical increase in relative abundance in the BRD-affected animals compared to the healthy animals (Fig. 1c). Interestingly, the prevalence of *Psychrobacter* and *Moraxella* was 89.45% and 91.46% in the apparently healthy samples (*n* = 199), respectively. In comparison, the prevalence of these bacteria in BRD-affected calves (*n* = 201) was 90% for *Psychrobacter* and 91.54% for *Moraxella*.

**Fig. 2:**
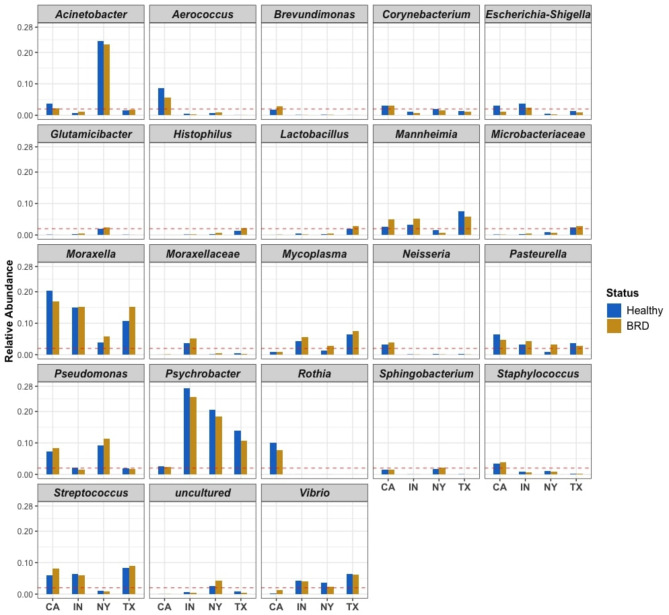
Genera from dairy cattle nasal swabs with an average relative abundance > 2% (red dashed line) in at least one health group (BRD-affected and apparently healthy cattle) from at least one of the four states. If only one group (apparently healthy or BRD) surpassed the 2% threshold (red dashed line), then both groups were reported

The nasal microbial communities exhibited both similarities and differences in composition among various farms and between BRD-affected and apparently healthy groups. Genera such as *Moraxella*, *Psychrobacter*, and *Pasteurella* were abundant, yet variable in abundance, in all farms and disease statuses. Specifically, the relative abundance of *Psychrobacter* was numerically higher in the apparently healthy group compared to the BRD-affected group across different farms (Fig. 2). In contrast, the relative abundances of *Moraxella* and *Pasteurella* was numerically higher or lower in the BRD-affected group, depending on the farm. Notably, only in NY was the abundance of *Pasteurella* numerically increased in relative abundance in the BRD-affected samples compared to the healthy group (Fig. 2). Furthermore, the relative abundances of *Histophilus*, *Mannheimia*, and *Mycoplasma* varied depending on disease status and farm. *Mycoplasma* abundance varied depending on disease status and farm. *Histophilus* showed higher relative abundance in TX, particularly with numerically higher abundance in the BRD-affected group (2.15%). BRD-affected animals exhibited a higher numerical relative abundance of *Mannheimia* and *Mycoplasma* compared to the apparently healthy group in three of the farms. *Lactobacillus* generally had a low relative abundance. The genera *Acinetobacter* dominated the nasal microbiome specifically in the NY samples. Lastly, the presence of *Pseudomonas* was abundant in the CA and NY samples, with BRD-affected animals having higher relative abundance than the apparently healthy group, while this genus was less abundant in IN and TX (Fig. 2).

### Differentially abundant taxa in the calf nasal cavity

**Fig. 3:**
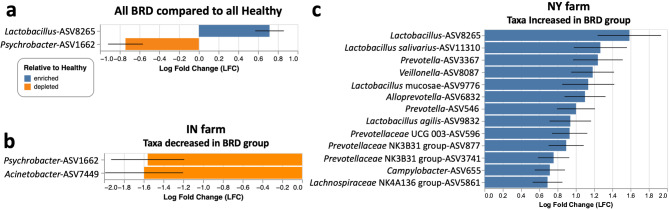
Differentially abundant taxa in between all the BRD-affected and apparently healthy animals (**a**) in the samples collected from IN (**b**), and in the samples collected from NY (**c**)

ANCOMBC analysis was used to identify differentially abundant taxa between BRD-affected and apparently healthy in the different farms. For this analysis, only ASVs with a sequence count greater than 50 counts across all samples were selected. Results revealed that when including all the BRD calves in the study, BRD calves had significant higher abundance of one ASV classified as *Lactobacillus* and lower abundance of one ASV classified as *Psychrobacter* compared to the apparently healthy calves 82 (*P* < 0.05, Fig. 3a). When the samples were divided by farm, the BRD-affected calves sampled from the IN farm had significantly lower abundance of one ASV identified as *Psychrobacter* and one ASV classified as Acinetobacter compared to the healthy group (*P* < 0.05, Fig. 3b). Additionally, a total of 13 different ASVs were significantly increased in the BRD-affected group from the NY farm compared to the apparently healthy group (*P* < 0.05, Fig. 3c). From these ASVs, two of them were classified as *Prevotella*, three were identified as members of the *Prevotellaceae* family and four were classified as *Lactobacillus* species. No other significant differential abundant taxa were detected from the CA and TX farms.

### Relative abundance of BRD pathogens in the dairy calf nasal cavity

Samples were divided by farm to assess the relative abundance of the four BRD-pathobionts and other bacterial genera associated with BRD outcomes (*Biberstenia trehalosi*, *Mycoplasma dispar*, also known as *Mesomycoplasma dispar*, *Mycoplasma bovirhinis*, also known as *Mycoplasmopsis bovirhinis*, and *Trueperella pyogenes*) among BRD-affected and apparently healthy calves (see to Additional File 1: Figure S1) The genus *B. trehalosi* was detected in all farms and disease statuses, with a relative abundance < 1%, except for TX, where it reached 6% solely in apparently healthy animals. *H. somni* and *M. haemolytica* had a relative abundance of < 1% in CA, IN, NY samples, regardless of disease status, except for TX, where *H. somni* was more abundant in BRD-affected animals (2%), and *M. haemolytica* had a numerically higher relative abundance in apparently healthy animals (5.4%). The relative abundance of *M. dispar* varied among farms, with IN, TX, and NY showing higher abundance in the BRD-affected group (> 1%), while CA had < 1% regardless of disease status. *M. bovirhinis* exhibited different relative abundance among farms but with similar values between apparently healthy and BRD-affected animals. *P. multocida* was the most prevalent BRD-pathobiont across all samples, with an average abundance of 3.70%, regardless of disease status. In IN (4.38%) and NY (3.34%) samples, BRD-affected animals exhibited numerically higher relative abundance of *P. multocida*. Among the farms, TX showed higher combined relative abundance of bacteria associated with BRD (> 15%), while all farms had a combined relative abundance of BRD associated bacteria of greater than 2%, indicating that the relative prevalence and abundance of these pathobionts is high (see Additional File 1: Figure S1).

### Dairy calf nasal microbiome alpha diversity

**Fig. 4:**
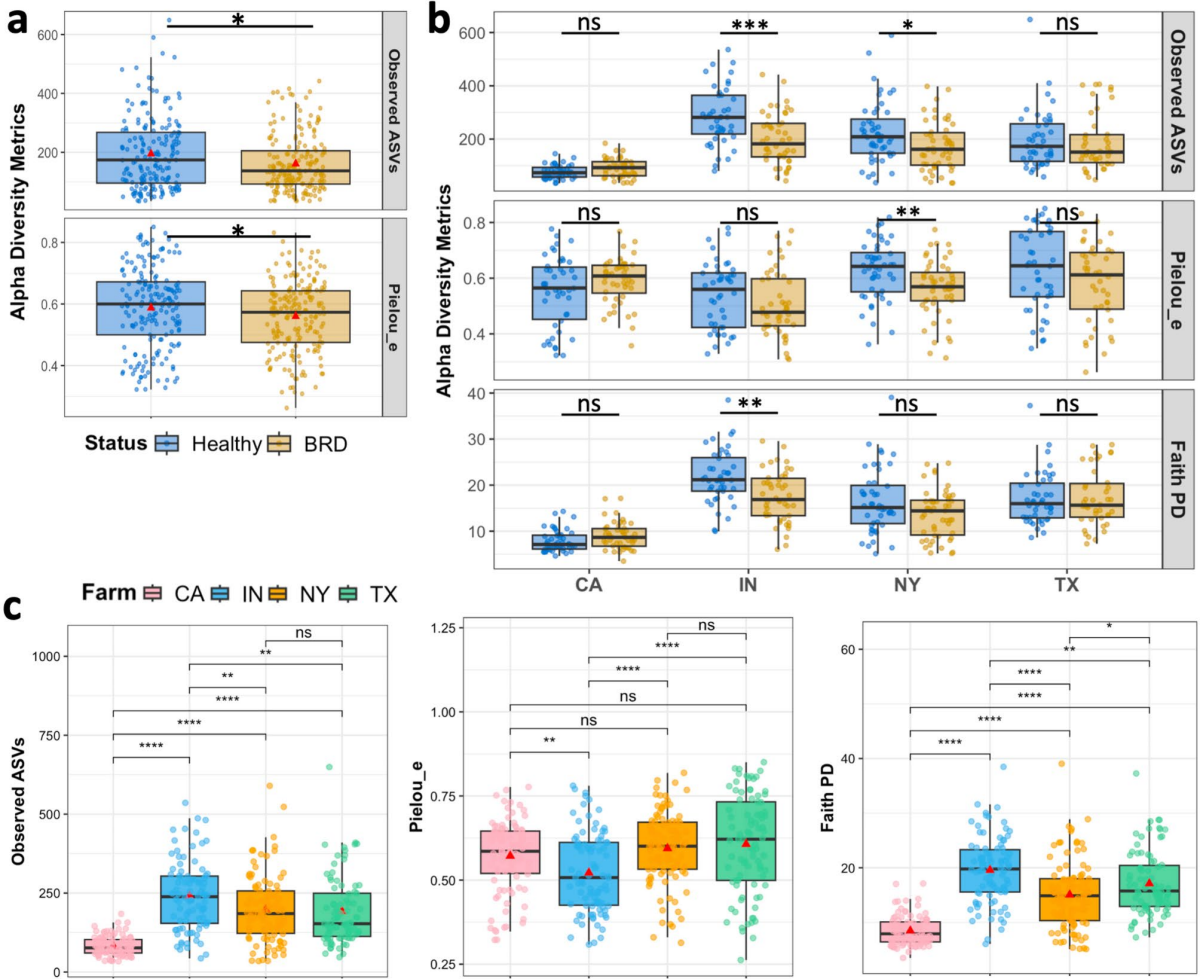
Alpha diversity metrics between apparently healthy and BRD-affected calves (**a**), separated by farm (**b**) and between all the farms **(c).** Red triangles represent the group mean

A total of 22,198,770 sequences, classified into 12,072 ASVs, were identified in DNA extracted from 404 dairy nasal samples collected from CA, IN, NY, and TX. After the denoising step (DADA2) and removal of *Pseudoalteromonas* from the samples, two samples were lost (402), leaving a total of 17,043,985 sequences classified in 12,051 ASVs. Samples were rarified to 12,237 sequences per sample, resulting in 10,809 ASVs that were used to quantify nasal microbiome alpha and beta diversity. The nasal microbiome richness measured through Observed ASVs, and evenness predicted by Pielou_e metrics, showed significant differences between apparently healthy and BRD-affected animals, irrespective of the farm, with BRD-affected animals exhibiting lower alpha diversity than the apparently healthy group (Fig. 4a). To assess potential effects of disease status on nasal cavity alpha diversity metrics, samples were separated by farm (Fig. 4b). In the IN farm, BRD-affected animals displayed significantly lower microbial richness and phylogenetic diversity compared to the apparently healthy group, with approximate decreases of 29% and 20%, respectively. Similar results were observed in NY, where BRD-affected animals had significantly lower microbial richness (23% decrease predicted by Observed ASVs) and a 10% decrease in nasal microbial community evenness. The farms from CA and TX exhibited no statistical difference between BRD-affected animals and their apparently healthy group for microbial richness (*P* > 0.05, Fig. 4b).

Additionally, nasal microbiome richness was mostly significantly different among the farms except for NY compared to TX. All farms showed significantly different phylogenetic relationships within the nasal cavity (Fig. 4c). Microbiome community evenness differed significantly between IN compared to CA, NY and TX (Fig. 4c). Interestingly, IN had the highest number of microbial species identified in the nasal cavity (Observed ASVs mean: 241), contrasting with CA having an Observed ASVs mean of 83. Additionally, phylogenetic diversity in the IN farm was the highest among farms, with a mean of 19.6 (predicted by Faith PD), while CA samples had the lowest phylogenetic diversity mean, 8.5, compared to the other farms.

### Dairy calf’s nasal microbiome beta diversity

**Fig. 5:**
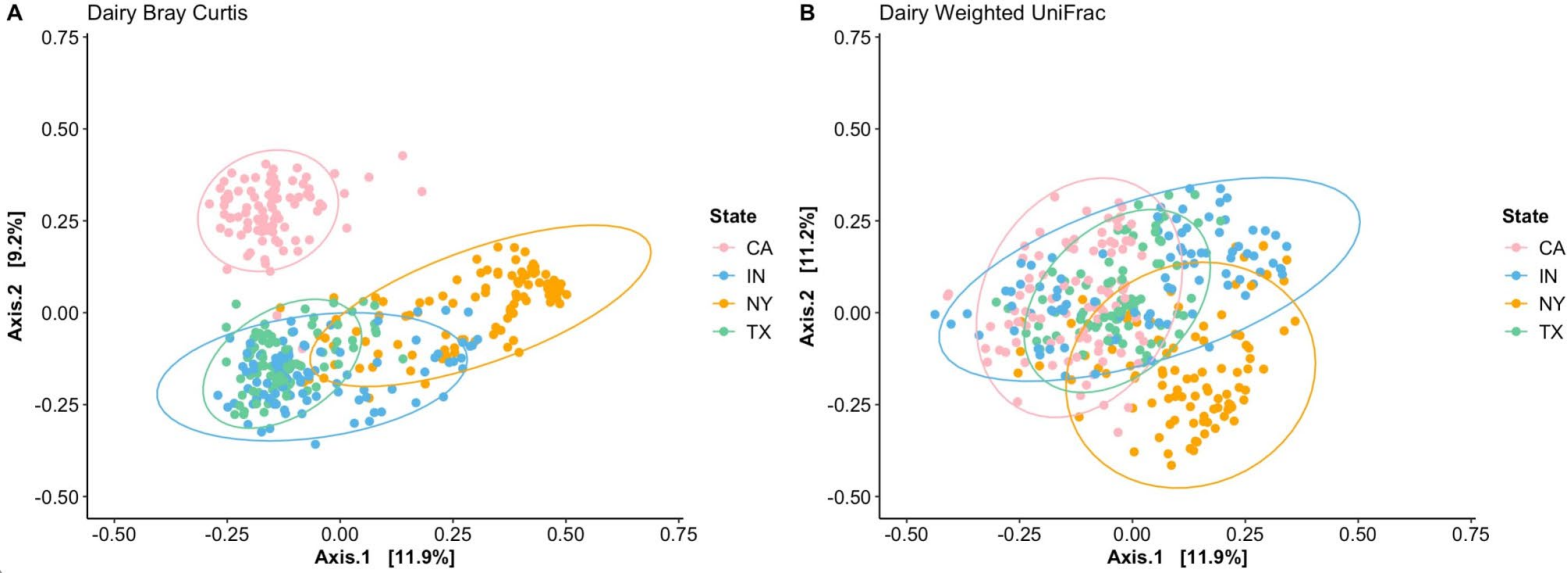
Beta diversity between apparently healthy separated by farm predicted by Bray-Curtis Dissimilarity (**a**) and Weighted UniFrac (**b**)

Bacterial community structure or the distance between BRD-affected and apparently healthy animals was not significantly different, as predicted by Bray-Curtis dissimilarity and Weighted UniFrac. Besides disease status, farm had a significant effect on nasal bacterial structure predicted by Bray-Curtis dissimilarity (F_1,367_ = 27.972, R2 = 0.187, *P* = 0.001, Fig. 5a) and Weighted UniFrac (F_1,367_ = 23.411, R2 = 0.161, *P* = 0.001, Fig. 5b). According to these results, when analyzing bacterial community structure without considering phylogenetic relationships (using Bray-Curtis dissimilarity), samples from CA displayed a dissimilar community structure compared to those from IN, NY, and TX (*P* < 0.001). However, with the inclusion of phylogenetic relationships, no clear pairwise separation was observed among the farms based on the PCoA plots (Fig. 5b). Finally, a dispersion test was conducted to identify the dispersion of each sample from the group centroids (apparently healthy and BRD-affected) to identify an overall disease status effect in the nasal beta diversity. This test revealed that the dispersion of BRD-affected and healthy samples from the group centroids was significantly different for the two beta diversity metrics (Dispersion Weighted UniFrac: *P =* 0.001, Dispersion Bray-Curtis: *P* = 0.002).

### Prevalence and quantification of BRD-pathobionts in the calf nasal cavity

**Fig. 6:**
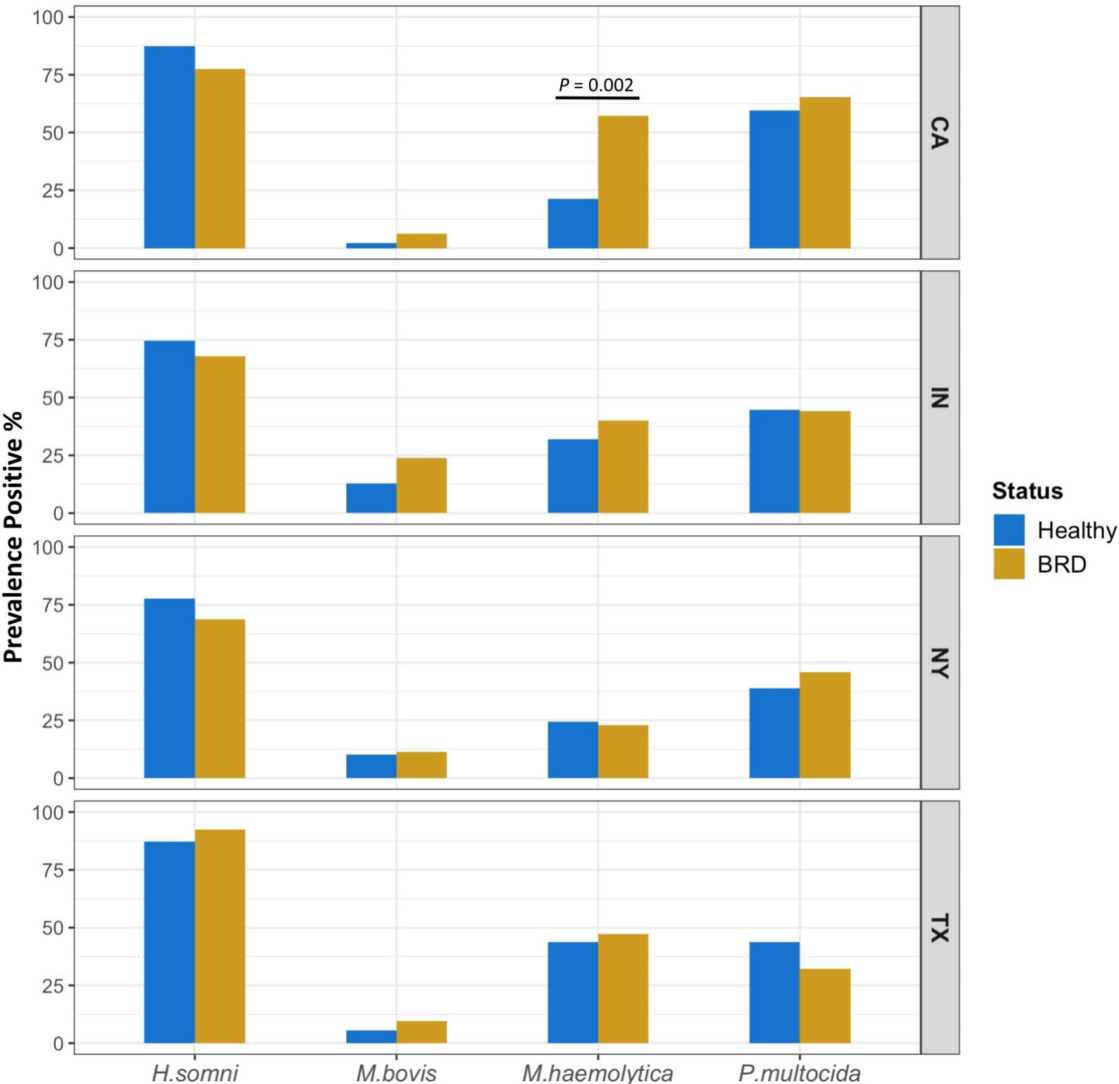
Prevalence of the BRD-pathobionts between apparently healthy and BRD-affected dairy calves sampled from CA, IN, NY, and TX farms. Prevalence values represent only the calves that tested positive (present) for each bacterium

Quantification of the four BRD-pathobionts, *H. somni*, *M. bovis*, *M. haemolytica*, and *P. multocida*, was conducted via qPCR using DNA extracted from the calf nasal swabs. Results from this study indicated that the bacterium *H. somni* is the most prevalent BRD-pathobiont regardless of the farm, with approximately 70% of the samples testing positive for the bacterium (Fig. 6), followed by *P. multocida* and *M. haemolytica*. *H. somni* was more prevalent in apparently healthy animals, except in the TX samples. *M. bovis* was the least prevalent bacterium, with a prevalence of less than 25% among all farms and regardless of disease status. Additionally, differences in the prevalence of BRD-pathobionts were observed among the farms and disease status. The prevalence of *M. bovis* and *M. haemolytica* was numerically higher in BRD-affected animals from CA, IN, and TX, except for samples collected from NY. However, only the prevalence of *M. haemolytica* in the samples collected from CA was significantly different between BRD-affected and the apparently healthy group (*P =* 0.002); no other significant differences were detected in the BRD-pathobiont prevalence between disease statuses and the different farms (Fig. 6).

**Fig. 7:**
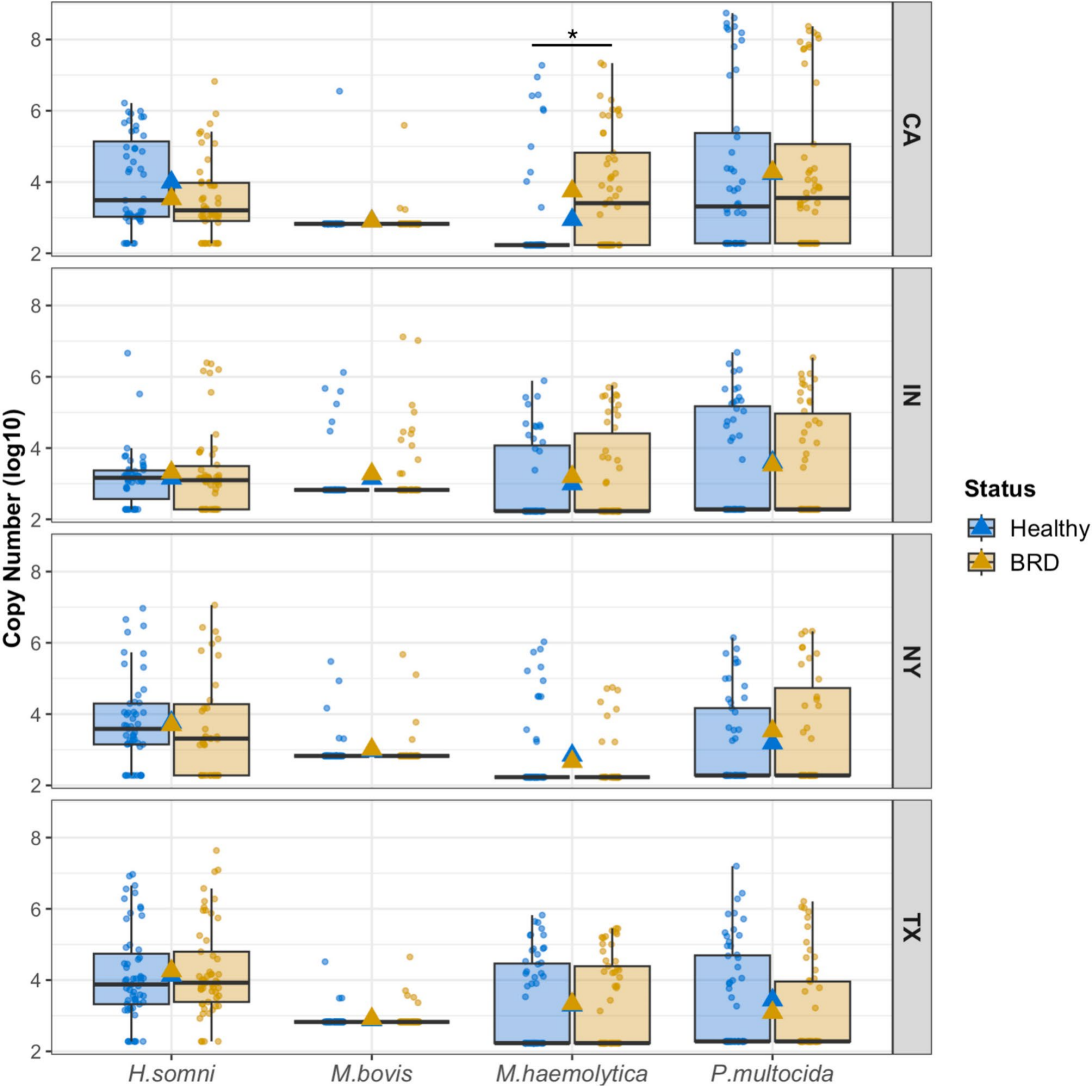
BRD-pathobiont abundance (log_10_) between disease status and divided by farm. Gold and blue triangles represent the group mean

The average abundance of *H. somni* among the farms and disease status was approximately 3.73 log_10_ per sample, *M. bovis* was 2.99 log_10_ per sample, *M. haemolytica* was 3.12 log_10_ per sample, and *P. multocida* was 3.61 log_10_ per sample. When separated by farm and disease statuses (Fig. 7), the abundance of *H. somni* and *P. multocida* ranged between 3 and 4 log_10_, while the abundance of *M. bovis* and *M. haemolytica* ranged between 2 and 3 log_10_. Statistical differences were identified in the abundance of *M. haemolytica* from the CA samples. Specifically, BRD-affected animals had higher abundance of *M. haemolytica* (copy number log_10_: 3.74 ± 1.62) compared to the apparently healthy animals (copy number log_10_: 2.94 ± 1.50), with no other significant differences detected.

A relative abundance test was used to discern the abundance of each BRD-pathobiont (*H. somni*, *M. bovis*, *M. haemolytica* and *M. bovis*) relative to the total nasal bacterial community and to the sum of the four BRD pathobionts. Differences in the relative abundance of *M. bovis* and *P. multocida* compared to the total bacterial abundance, based on the 16 S rRNA gene abundance per sample, were identified in the NY samples (see Additional File 1: Figure S2a, b). On the other hand, samples from CA showed that the relative abundance of *M. haemolytica* compared to the total abundance of the four pathobionts was significantly higher in the BRD-affected group compared to the healthy group (see Additional File 1: Figure S2c). No significant differences in the abundance of the BRD-pathobionts and their relative abundance were identified in the samples collected from IN and TX farms. Quantification of the total nasal bacterial abundance in the samples collected from CA, IN, NY, and TX was performed via qPCR. In the study, the total bacterial abundance determined by the abundance of the 16 S rRNA gene was significantly different between the farms (see to Additional File 1: Figure S3a), except for the bacterial abundance between IN compared to NY. Interestingly, CA samples had a higher mean bacterial abundance than the other farms. Lastly, differences in the total bacterial abundance between BRD-affected and apparently healthy were detected in the NY samples, with healthy animals having significantly higher total bacterial abundance than the BRD-affected animals (see to Additional File 1: Figure S3b). No other significant differences in the total bacterial abundance were detected in the study.

### Prevalence and quantification of *P. multocida* and *M. haemolytica* pathogenic serotypes

Nasal swabs collected from IN (*n* = 83) and NY (*n* = 74) farms underwent quantification of *P. multocida* serotype A and *M. haemolytica* serotype A1 and A6 to discern differences in abundance between apparently healthy and BRD-affected calves. In the IN samples, the prevalence of *M. haemolytica* A1 was approximately 58% in the apparently healthy group and 63% in the BRD-affected group, while the prevalence of *M. haemolytica* A6 was 69% in the apparently healthy and 57% in the BRD-affected group. The prevalence of *P. multocida* serotype A was closely similar between apparently healthy animals (67%) and BRD-affected animals (64%) (see Additional File 1: Figure S4). Unfortunately, no significant differences were observed in the abundance of *P. multocida* and *M. haemolytica* pathogenic serotypes based on disease status (*P* > 0.05). In the case of the NY samples, the prevalence of *M. haemolytica* A1 (44% and 31%) and A6 (59% and 34%), as well as *P. multocida* A (49% and 46%), had a higher prevalence in apparently healthy animals compared to the BRD-affected animals, respectively (see Additional File 1: Figure S4). The IN samples had a higher average abundance of *M. haemolytica* A1 (3.38 log_10_ per sample) than A6 (2.74 log_10_ per sample), while NY samples exhibited a similar abundance between both serotypes, approximately 2.69 log_10_ per sample (Table 1). The abundance of *P. multocida* serotype A was similar regardless of farm and disease status (Table 1). No significant differences were detected in the abundance and prevalence of each serotype between disease statuses (*P* > 0.05).

**Table 1:** Abundance of the *M. Haemolytica* and *P. Multocida* pathogenic serotypes in IN and NY and between disease status

Farm	Serotype	Status	*n*	Mean	SD	SE	25%*	75%*
IN	Mh-A1	Healthy	36	3.31	1.34	0.22	2.86	3.76
		BRD	47	3.46	1.45	0.21	3.03	3.88
	Mh-A6	Healthy	36	2.78	0.42	0.07	2.64	2.93
		BRD	47	2.69	0.46	0.07	2.55	2.82
	Pm-A	Healthy	36	3.75	1.30	0.22	3.31	4.19
		BRD	47	3.61	1.30	0.19	3.23	3.99
NY	Mh-A1	Healthy	39	2.80	1.08	0.17	2.45	3.15
		BRD	35	2.61	0.91	0.15	2.30	2.93
	Mh-A6	Healthy	39	2.78	0.56	0.09	2.60	2.96
		BRD	35	2.57	0.56	0.10	2.38	2.77
	Pm-A	Healthy	39	3.38	1.20	0.19	2.99	3.77
		BRD	35	3.42	1.33	0.22	2.96	3.87

*25% and 75% quartile, Mh-A1 = *M. haemolytica* serotype A1, Mh-A6 = *M. haemolytica* serotype A6, Pm-A = *P. multocida* serotype A

## Discussion

Bovine respiratory disease (BRD) is an ongoing health and economic issue in the dairy and beef cattle industries, and it is caused by multiple factors such as predisposing, environmental, and epidemiological factors [2]. Diagnosis and treatment have proven challenging for producers, as BRD diagnosis predominantly relies on observation of visual clinical signs, yet not all animals exhibit these signs [7]. Previous studies have recognized the potential of using the bovine nasal microbiome to aid in BRD identification [9–11, 13, 14]. Consequently, this study sought to characterize the nasal microbiome community in dairy calves displaying BRD clinical signs and their apparently healthy counterparts, utilizing samples from different dairy farm locations in the USA (CA, IN, NY, and TX). The goal was to identify potential differences in the commensal and BRD-pathobiont microbiomes between healthy and BRD-affected calves. Our results indicate that nasal alpha diversity emerged as an indicator of disease status, but this effect was not consistent on all farms. The nasal microbial community structure remained similar regardless of disease status. Nasal microbiome diversity differed by farm. In some cases, specific members of the nasal microbiome were linked to disease status. Moreover, among the BRD pathobionts, *H. somni* exhibited the highest prevalence across all farms, followed by *P. multocida*, *M. haemolytica*, and *M. bovis*. However, no significant differences in the abundance of BRD pathobionts were observed between apparently healthy and BRD-affected cattle, except for *M. haemolytica* in samples from CA. Additionally, no significant difference in the abundance of specific pathobiont serotypes (*M. haemolytica* A1 and A6, and *P. multocida* serotype A) were detected between BRD and apparently healthy groups. This study proves valuable for the BRD field, particularly in the dairy cattle industry, by identifying core members of the calf nasal microbiome community regardless of the farm and potential traits that could aid in distinguishing disease status.

### Nasal microbiome taxonomy composition revealed prevalent members of the dairy calf nasal microbiome

Prevalent members of the dairy calf microbiome were identified regardless of disease status and farm location. In this study, the nasal microbiome was dominated by two members of the *Moraxellaceae* family, *Moraxella* and *Psychrobacter*, with *Pasteurella* present at a lower relative abundance. *Psychrobacter*, a known member of the bovine nasal and nasopharyngeal microbiomes, has been identified in various animal microbiomes, but also in non-host environments like seawater, sea ice, marine sediment, glacial ice, and permafrost soil. While *Psychrobacter* species have been recovered from mammalian hosts, including marine mammals, birds, and fish, their capability to cause disease is rare, and the factors influencing infection remain unclear [27]. This microbe has occasionally been associated with BRD occurrences [9, 28–30]. But studies have also suggested that it possesses antagonistic effect on *Mycoplasma* abundance [9, 31]. While *Psychrobacter* inhibitory capacities have not been tested against BRD-pathobionts, one study tested the growth inhibitory capacities of *Psychrobacter* in gram negative human pathogens (*Serratia marcescens*, *Pseudomonas aeruginosa* PAO1, *Vibrio parahemolyticus* and *V. vulnificus*) [32]. The authors observed that in vitro treatment of *Psychrobacter* inhibits the biofilm formation. Additionally, in the case of *P. aeruginosa* PAO1, *Psychrobacter* inhibits the growth, motility and biofilm formation in a concentration dependent manner, highlighting the inhibitory capacities of this microbe against gram negative bacteria [32]. Intriguingly, in our samples *Psychrobacter* was one of the most prevalent and abundant genera in the dairy nasal samples, but it only had numerically higher relative abundance in apparently healthy animals compared to the BRD-affected group.

The relative abundance of *Moraxella* varied by farm, particularly with numerically higher abundance (10–20%) in CA, IN, and TX. The role of *Moraxella* in bovine respiratory disease is still unclear and requires further investigation. Some studies have identified this microbe as a commensal member of the cattle upper respiratory microbiome [27, 33], while others have shown that *Moraxella* was associated with BRD development [31, 34, 35], with high abundance immediately following transport in heifers that subsequently developed BRD [9–14] or present in the nostrils of BRD-affected cattle [9].

### Nasal alpha diversity differed between apparently healthy and BRD-affected dairy calves, in some farms

In this study, the nasal microbiome in the BRD-affected animals presented a lower number of observed ASVs (richness) and Pielou Evenness, irrespective of the farm. After separating the samples by farm, BRD-affected calves in IN and NY had lower alpha diversity metrics than apparently healthy animals. Reduction in alpha diversity, particularly microbial richness and evenness, has been reported in the upper respiratory tract of BRD-affected cattle in previous studies [11, 36, 37]. A study by Centeno-Martinez et al. (2022) [9] also reported a 20% decrease in nasal microbiome richness in BRD-affected Holstein beef steers, similar to our findings here where microbial richness (Observed ASVs) in BRD-affected animals from IN and NY decreased by 29.8% and 24%, respectively. Thus, apparently healthy animals harbored a greater number of microbial species within the nasal community compared to those affected by BRD. Moreover, BRD-affected animals from IN experienced an approximate 20% decrease in phylogenetic diversity compared to the apparently healthy group. Similar trends were noted in Centeno-Martinez et al. (2022) [9], where the BRD-affected group exhibited an 11% decrease in phylogenetic diversity. Previous studies have associated higher microbiome diversity with ecosystem stability and resistance to pathogen colonization [38, 39]. Consequently, the higher observed microbial richness and phylogenetic diversity in apparently healthy animals, particularly in IN and NY, may confer a more stable community and potential resistance to pathogen colonization [39, 40]. However, it is crucial to note that these differences are farm specific. Additional confounding factors may exist, such as, age, time of sampling, effect of housing (individual/groups), stage (pre-weaned/post-weaned), and breed.

### Similar BRD-pathobiont abundance and serotypes in BRD-affected and apparently healthy animals

Development of BRD has been linked to the presence of multiple pathogens, including the bacterial species *H. somni*, *P. multocida*, *M. haemolytica*, and *M. bovis* [16, 41], in the upper and lower respiratory tract, and specifically lung tissue samples from BRD mortalities [16, 41]. Due to their significance in BRD development, various studies have focused on quantifying the abundance of these bacteria in the upper respiratory tract, aiming to develop BRD diagnostic tools and identify differences between cattle diagnosed with BRD and healthy cattle [9, 10, 13, 14, 42–45]. Interestingly, in the current study, no significant differences were detected in the prevalence or abundance of pathobiont between farms and disease status in dairy calves, except at one farm (CA) for one pathobiont (*M. haemolytica*). Among all the samples, the average abundance of the BRD-pathobionts in the nasal cavity is between 10^2^ and 10^4^ per sample (depending on the farm), similar to previous studies [9, 43].

As a secondary test to identify differences in nasal pathobiont carriage for disease detection, an abundance analysis of the most pathogenic serotypes within *M. haemolytica* (serotype A1 and A6), and *P. multocida* (serotype A) [19–21, 46], was conducted on samples collected from IN and NY. Nevertheless, the abundance of these microbes in the calf nasal cavity was similar. Results from this study indicate that the abundance of BRD-pathobionts in the nasal cavity is not significantly different between BRD-affected and apparently healthy calves, and these results are consistent regardless of the farm.

It is unclear why the prevalence and abundance of BRD-pathobionts in the nasal cavity of dairy calves is not higher in in dairy calves with BRD than in healthy calves. It is possible that the age of the animal is important to the use of the nasal pathobionts to predict disease. The animals in the current study were mostly less than two months old. Our previous study in 6–7 month-old Holstein beef steers showed clear differences in pathobiont abundance between healthy and BRD animals [9]. In another study, the authors observed higher abundance of the *H. somni*, and *M. haemolytica* in the BRD-affected calves compared to the healthy calves; however, this study used bronchioalveolar lavage rather than nasal samples and the dairy calves were 1–5 months old [47]. Few other studies of the young dairy calf nasal microbiome are available. Previously, we performed a short study on the progression of the dairy calf nasal microbiome from 1 to 4 weeks of age and found little relationship between the relative abundance of BRD pathobionts and disease status [48]. Thus, more research needed to identify how the dairy nasal microbiome differs between BRD-affected and apparently healthy calves and how this relationship changes as animals grow older. Alternatively, there are also many other potential confounding factors between studies in beef and dairy cattle.

### Nasal microbiome taxonomy is significantly altered by geographic location

A notable geographic effect was observed in shaping the nasal microbiome community, aligning with results from Chai et al. (2022) [29] in their investigation of the bovine respiratory microbiome across different locations in China and Canada. The authors compared nasopharyngeal samples collected from two geographic locations: samples from Canada had a higher relative abundance of the bacterial species *Burkholderia* while the two cities in China had a higher relative abundance of *Moraxella catarrhalis*, *Psychrobacter* sp., *Corynebacterium* species, and *Mycoplasma conjunctivae.* Additionally, the nasopharyngeal microbiome of the Canadian samples was mainly composed of *Proteobacteria* members while the two cities in China were mostly composed of *Proteobacteria* and *Firmicutes.* Even though these studies targeted the nasopharyngeal microbiome community, a similar geographic effect on the bovine nasal microbiome community has been detected in previous studies, indicating the importance of including the geography during data analysis [31, 49]. However, these studies did not mention the impact of farm management, diet, breed, or other factors that could be different between farms in addition to the influence of geography.

### Nasal bacterial community structure is similar between apparently healthy and BRD-affected dairy calves

Analysis of nasal microbial structure, or beta diversity, provides insight into the similarity or dissimilarity of the microbiome community between apparently healthy and BRD-affected animals. Dysbiosis, or disruption in microbial community structure, is sometimes associated with disease incidence and progression [50, 51]. Therefore, our study aims to identify any nasal microbiome disruption due to disease status. Based on our findings, the nasal microbiome community structure did not differ between BRD-affected and apparently healthy animals. Additionally, differences were observed between farms, irrespective of disease status. Particularly, CA samples exhibited a dissimilar microbiome community structure compared to the other farms, as predicted by Bray-Curtis dissimilarity. Similar to other studies, geography has been detected to play a role in determining the microbiome composition [25, 50]. In a human study, geographic location (14 districts within one province in China) had the strongest association with the human gut microbiome composition [50]. The authors used a machine model method to predict an association between the host individual’s metabolic health (disease classification) and their gut microbiome data, resulting in low prediction accuracy. However, when samples were divided according to geographic location, disease classification substantially improved. These results underscore the importance of accounting for geographic effects and avoiding extrapolation of results to prevent inaccurate predictions.

Despite the strengths of this study, experimental limitations may limit our ability to identify differences between healthy and BRD animals, many of which are common to most BRD studies. First, we collected nasal swabs which do not completely represent the lung microbiome [27]. Second, we focused on bacteria, while the presence of viruses and fungi have also been linked to disease development [41, 52–55]. Third, animals were only visually classified as BRD or healthy, which can misidentify the BRD status of animals [8]. Fourth, animals were managed differently in different farms, including housing, which could alter BRD transmission between animals [56]. Fifth, we did not measure the immune status of the animals, which may allow some animals to withstand pathogen invasion of the mucosal surface [18]. On the other hand, we feel that the strengths of this study were the number of samples (about 400), sampling five independent farms, quantifying the abundance of BRD pathobionts by qPCR, and use of nasal swab samples that could be easily collected by any farm staff. Notwithstanding the limitations of the study and contrary to our initial hypothesis, our study seems to make clear that BRD development in dairy calves is not indicated by an increase in pathobionts in the nasal passage or by an overall disruption or dysbiosis of the bovine nasal microbiome. Thus, further studies, like the current one, are needed to deepen the understanding of how additional animal factors (including immune status) influence the respiratory microbiome.

## Conclusions

BRD-affected calves from the farms in IN and TX had lower alpha diversity compared to their apparently healthy group. The bovine nasal bacterial community structure was different between farms, but not by disease status. This result indicates that farms, including potential confounding factors like age, environmental factors, housing, or breed may influence the nasal microbial structure. Contrary to our hypothesis, the abundance of the BRD-pathobionts and their serotypes was not different between BRD-affected and apparently healthy animals. Both groups share a similar pathobiont carriage, but their values were dependent on farm. Further research is essential to comprehend the temporal changes in the microbiome, including the impact of different farm management observed in this study.

## Materials and methods

### Cattle nasal swab collection

#### Animal Population

Nasal swabs were collected from five different dairy farms (CA, IN, NY, and TX) in the US from 2020 to 2022, with each animal being sampled once. Two dairy farms were sampled in NY located less than 10 miles apart from each other. These two farms were grouped together due to their geographic proximity and similarity in microbiome composition (Additional File 1, Fig. S6). Approximately 100 samples were collected from each farm, comprising 50 samples from visually healthy animals and 50 from BRD-affected animals. However, due to sample processing and missing information, some samples were not included in the study. Animal selection was carried out using the DART method as specified in Centeno-Martinez et al., (2022) [9]. The DART method employs visual clinical signs such as depression, appetite loss, respiratory character change, and rectal temperature (> 103 °F) as criteria [41]. Once an animal was visually identified as BRD-affected by the farm personnel following the DART method, one or two healthy calves were selected based on the absence of BRD visual clinical signs. Unfortunately, the measurement of rectal temperature > 103 °F was not always an indicator of disease, as some visually healthy animals also presented rectal temperature > 103 °F. Therefore, rectal temperature was not included as a factor to determined disease status. Information on the collected animal data for each of the farms is found in Table 2.

**Table 2:** Dairy farm data

Farm	Breed	Age	Housing	Stage	Temp/Sampling
CA^1^	Holstein	< 2 months	Individual hutches	Pre-weaned	> 37 °C
IN^2^	Holstein	1–4 months	Individual hutches (< 8 weeks old), group housing (> 8 weeks old)	Pre and post weaned	> 0 °C
NY^3^	Holstein	Calves and cows	Individual and group housed	Calves and fresh cows	21 °C
TX^1^	Jersey	< 2 months	Individual hutches	Pre-weaned	< 0 °C

^1^ Animals selected from the same farm and all of them were calves

^2^ Animals selected from the same farm and some samples were collected from cows (Cow samples = 6, calves samples = 91)

^3^ Samples collected from two different farms (Farm 1 = 70 samples, Farm 2 = 31 samples)

### Cattle nasal swab collection and DNA extraction

For the study, one double swab was collected from each animal sampled from CA, IN, NY, and TX. After collection, the samples were kept in the refrigerator at the farm facilities before being shipped to the lab. Samples were shipped within 5 days of collection and processed within 7 days of collection. Nasal swabs were processed following the protocol by Centeno-Martinez et al. (2022) [9]. Total DNA extraction was carried out using the DNeasy Blood and Tissue Kit (Qiagen, Germantown, MD, United Farms) following the protocol described by Centeno-Martinez et al. (2022) [9], adapted from Holman et al. (2015) [57]. The 16 S rRNA library pool was prepared using the extracted DNA to characterize the total bacterial community present in the nasal cavity following the Kozich et al. (2013) [58] protocol. PCR amplification was performed using AccuPrime Pfx SuperMix (Thermo Fisher Scientific MA, USA). PCR-grade water was used as the negative control, and a mock community containing 20 known bacterial DNA strains served as the positive control (20 Strain Even Mix; ATCC. MSA-1002TM). PCR amplification was verified using gel electrophoresis, and the amplified DNA was normalized using the SequalPrep Normalization Kit. A total of 5 µl of the normalized DNA per sample was combined into a pool and sent to the Purdue Genomic Core Facility for sequencing via Illumina MiSeq (2 × 250 paired-end).

### 16 S rRNA gene bioinformatic analysis

The raw sequences obtained from the 16 S rRNA gene sequencing were analyzed using Quantitative Insight Into Microbial Ecology (QIIME2) v.2022.8. The raw forward and reverse sequences were trimmed using DADA2 to obtain sequences with a quality > Q30 [59]. All sequences were clustered into Amplicon Sequence Variants (ASVs) with 100% similarity. Taxonomy was assigned using SILVA 138, 515/806 region database. An analysis of composition of microbiome with bias correction (ANCOM-BC) was performed to identify differentially abundant taxa between healthy and BRD animals in dairy farms. This method accounts for the underlying compositional characteristics of the microbiome community [60, 61]. For this step, the ASV table was filtered to remove ASVs with sequence counts  < 50 across all samples and a significant threshold of 0.05 was used to detect the significant differential abundant taxa.

To calculate alpha and beta diversity, the ASV table was rarefied to 12,237 sequences per sample. During subsampling, some samples were lost due to low sequence count (total dairy samples = 404, after rarefying = 368, Table 3). Alpha diversity, which measures community richness and evenness, was estimated in QIIME2 using Observed OTUs, Chao1 to determine richness, and Faith’s phylogenetic diversity (Faith PD) and Pielou Evenness to determine community evenness [62–67]. Beta diversity, indicating microbial community structure, was determined using the Bray-Curtis Dissimilarity Index and Weighted UniFrac (incorporates phylogenetic diversity) methods and plotted as principal coordinate analysis (PCoA) using RStudio [68]. To test community structure, a permutational multivariate analysis of variance test (PERMANOVA; *P* ≤ 0.05) was applied to the dairy samples using the function ‘adonis2’ from the vegan package [69]. Beta diversity was compared between BRD-affected and visually healthy animals across all different farms. To account for the farm effect, we included disease status as a between factor and farm as a within factor using the function ‘strata’. If a significant result was observed, a pairwise comparison analysis (*P* ≤ 0.05) using the function ‘pairwise.adonis2’ was applied. In addition, a dispersion test was performed to determine the distance of each sample from the group centroid (BRD or healthy) using the vegan package following a permutation test of multivariate homogeneity of group dispersion [69].

**Table 3:** Summary of the total nasal swabs data collection from four different dairy farms in the US before and after rarefication

Dairy Farm Samples Before Rarefying			Dairy Farm Samples After Rarefying		
Farm	Healthy	BRD	Farm	Healthy	BRD
CA (*n* = 96)	47	49	CA (*n* = 94)	46	48
IN (*n* = 97)	47	50	IN (*n* = 90)	44	46
NY (*n* = 101)	50	51	NY (*n* = 98)	48	50
TX (*n* = 110)	55	55	TX (*n* = 86)	44	42

### DNA extraction and sequencing controls analysis

To verify PCR amplification and sequencing accuracy, a positive control comprising 20 known bacterial DNA strains was included as a mock community (ATCC MSA-1002TM). The mock community was sequenced concurrently with the dairy samples. The raw mock community sequences were analyzed separately from the samples to assess amplification and sequence quality. Using QIIME2 v.2022.8, we compared the mock sequences with a reference file containing the 16 S rRNA gene sequences of the 20 known bacterial strains. The forward and reverse sequences were trimmed to retain sequences with a quality score > 30. To assess sequencing quality, we utilized the ‘evaluate_seqs’ function in QIIME2, aligning the observed sequences in the mock samples with the mock reference file and determining matches and mismatches.

In addition to the positive control utilized in the PCR amplification and sequencing stage, two types of negative controls were incorporated: empty tubes and PCR water. The empty tubes served as samples processed concurrently with the nasal swab samples during DNA extraction, representing the DNA extraction kit negative control. This facilitated the identification of potential contaminants in the reagents used for extraction, ensuring the integrity of the extracted DNA. The PCR water served as the negative control during the PCR amplification step. Both empty tubes and PCR water underwent sequencing simultaneously with the positive control and dairy samples. Raw sequences obtained from the empty tubes and PCR water were analyzed separately from the positive control and dairy samples using QIIME2, following the aforementioned procedure. Similarly, the forward and reverse sequences were trimmed to eliminate low-quality sequences (< 30). Finally, taxonomy was assigned using SILVA 138, 515/806 region database.

Sample contamination during DNA extraction and sequencing can stem from various sources, such as sample collection, laboratory equipment, DNA extraction reagents, or contaminated water. To assess the potential presence of contaminants in the extracted DNA, we followed the protocol outlined by Centeno-Martinez et al., (2022) [9], utilizing the observed sequences in the mock community and the negative controls (empty tubes and PCR water). If an amplicon sequence variant (ASV) was identified as contamination in the samples, it was subsequently eliminated. In this study, one ASV classified as *Pseudoalteromonas* was identified as a contaminant and consequently removed from the samples due to its presence in both the DNA extraction negative controls (empty tubes) and the dairy nasal swabs. Following the removal of *Pseudoalteromonas*, three samples were excluded due to the high abundance of these ASVs in the samples (see Additional File 1: Figure S5).

### Quantification of BRD-pathobionts in the cattle nasal cavity

Extracted DNA from the BRD-pathobionts *P. multocida*, *H. somni*, *M. haemolytica*, and *M. bovis* were used to construct a qPCR standard curve to quantify its abundance in the cattle’s nasal cavity following the protocol by Centeno-Martinez et al. (2022) [9]. DNA was extracted from pure isolates of *P. multocida*, *H. somni*, and *M. haemolytica* obtained from the Indiana Animal Disease Diagnostic Laboratory (ADDL) at Purdue University, and DNA from *M. bovis* was obtained from *M. bovis* strain 25523 (ATCC). Primers utilized to target the presence of the BRD-pathobionts are listed in see Additional File 1: Table S1. To construct the qPCR standard curve, PCR assays were conducted to generate the BRD-pathobionts gene amplicons in a 50 µl volume comprising 25 µl of iTaq™ Universal Probes Supermix (BioRad, CA, USA), 12 µl Primer/Probe mix (refer to see Additional File: Table S1) with a primer concentration of 0.3 µM and a probes concentration of 0.1 µM, 10 µl of nuclease-free water, and 2.5 µl of DNA template. PCR assays were carried out using the Eppendorf Mastercycler Gradient Model 533, with cycling conditions aligned with the protocol by Centeno-Martinez et al. (2022) [9]. PCR-grade water served as the negative control, and PCR amplification was verified through gel electrophoresis. Subsequent to obtaining the gene amplicons for each of the BRD-pathobionts, the amplicons were cleaned and purified using the Monarch PCR and DNA cleanup kit (New England BioLabs, MA, USA).

A 9-fold serial dilution (10^8^ to 10^0^) was generated for each of the BRD-pathobiont amplicons to generate the qPCR standard curve. The qPCR technical replicate assays were performed following Centeno-Martinez et al. (2022) [9] protocol. In brief, a total volume of 20 µl was prepared for each BRD-pathobiont assay. Each reaction consisted of 10 µl of iTaq Universal Probes Supermix (BioRad, CA, USA), 5 µl of Primers/Probes with the same concentration as mentioned earlier, and 5 µl of each BRD-pathobiont amplicon. All qPCR assays were executed in the CFX96 Real-Time System Thermal Cycler (BioRad, CA, USA), and the cycling conditions for each BRD-pathobiont were consistent with those described in Centeno-Martinez et al., (2022) [9]. The standard curve was constructed by linear regression of the technical triplicate average cycle quantification (Cq) for each sample and log10 amplicon copies/µl from each dilution. One dilution employed for generating the standard curve for each bacterium served as the positive control, and PCR-grade water was used as the negative control in each of the qPCR triplicates.

### Quantification of the total bacteria abundance in the cattle nasal cavity

Quantification of the total bacteria found in the cattle’s nasal cavity was performed by targeting the bacterial 16 S rRNA gene. A pool of extracted DNA from various nasal swabs served as the nucleic acid template for the PCR reaction. The 16 S rRNA gene PCR was carried out using the Eppendorf Mastercycler Gradient Model 533. Two bacteria-specific primers, 8 F and 1492R, were selected to target the 16 S rRNA gene, which was subsequently utilized as the qPCR nucleic acid template [70]. PCR assays were conducted in a 50 µl volume reaction, comprising 42.5 µl of AccuPrime™ Pfx SuperMix (Thermo Fisher Scientific, MA, USA), 2.5 µl of each primer (8 F and 1492R), 1.5 µl of nuclease-free water, and 1 µl of DNA template. PCR cycling conditions and primer concentrations were set as per Kozich et al. (2013) [58]. A mock community (20 Strain Even Mix 138 Genomic Material, ATCC. MSA-1002TM) and PCR-grade water were included as the positive and negative controls, respectively, during the PCR step. PCR amplification was verified through gel electrophoresis. Finally, the 16 S rRNA gene amplicons were purified using the Monarch PCR and DNA Cleanup kit (New England BioLabs, MA, USA).

Amplicons of the 16 S rRNA gene underwent a 9-fold dilution (10^8^ to 10^0^) to generate the standard curve. qPCR assays were conducted in a 20 µl total volume, consisting of 10 µl LightCycler 480 SYBR Green I Master (Thermo Fisher Scientific, PA, USA), 5 µl of primers (universal primers 1132 F and 1108R), and 5 µl of the 16 S rRNA amplicon. The conditions for 16 S rRNA qPCR and primer concentrations were in accordance with the protocol by Leigh et al. (2007) [71], and the assays were executed in a CFX96 Real-Time System Thermal Cycler (BioRad, CA, USA). Standard curve generation, along with the inclusion of positive and negative controls, followed the previously described procedure.

The quantification of BRD-pathobionts’ abundance in the nasal cavity relied on the prevalence of each bacterium. The cut-off limit of detection (LOD) value determined the classification of samples as positive (Cq value below LOD) or negative (Cq value above LOD) for each bacterium (see Additional File: Table S2). Additionally, the relative abundance of BRD-pathobionts was calculated by dividing the copy number of each bacterium by the 16 S rRNA gene copy number obtained per sample (labeled as BRel) and by the sum of the copy numbers of all four bacteria (PRel). This analysis allowed the identification of the relative abundance of each bacterium concerning the total bacterial community in the sample (BRel) and the relative abundance of each bacterium concerning the other BRD-pathobionts (PRel).

### Quantification of *M. haemolytica* and *P. multocida* pathogenic serotypes

We quantified and determined the abundance of BRD-pathobionts, specifically *M. haemolytica* serotype A1 and A6, and *P. multocida* pathogenic serotype A. In a prior study conducted by Sheets (2023) [72] and Wickware (2022) [73], DNA was extracted from *M. haemolytica* and *P. multocida* isolated from lung tissue samples collected from cattle necropsies. These isolates underwent whole-genome sequencing for genome identification, annotation, and serotyping confirmation. Utilizing *P. multocida* and *M. haemolytica* genome sequences as input, the authors predicted serotypes using a server and database created by Christensen et al. (2021, 2022) [74, 75]. Details regarding the *M. haemolytica* and *P. multocida* isolates chosen for the test are available in Additional File: Table S3. *P. multocida* predicted serotype A served as the target, and *P. multocida* predicted serotype D was included as the negative control. Additionally, *M. haemolytica* predicted serotype A1 and A6 were considered targets, while isolates predicted as A2 and A5 were utilized as negative controls. Our expectation was to develop BRD-pathogen-specific qPCR capable of distinguishing the presence of *P. multocida* (serotype A) and *M. haemolytica* (serotype A1 and A6) pathogenic serotypes from non-pathogenic serotypes: *P. multocida* serotype D and *M. haemolytica* serotype A2 and A5.

Using aseptic techniques, frozen isolates of *M. haemolytica* and *P. multocida* pathogenic and non-pathogenic subspecies were streaked on blood-agar medium (Tryptone Soya Agar with sheep blood, Thermofisher Scientific, Waltham, MA, USA). Incubation occurred in a microaerophilic chamber with 5% CO2 at 37 °C for 18 to 24 h. If growth was observed, a single colony was subcultured in liquid culture media containing 2.8% Brucella Medium Base (Thermofisher Scientific, Waltham, MA, USA) at 37 °C with agitation just below 200 rpm. Bacterial cells from the liquid culture were replated (blood agar medium) and subcultured in new liquid culture media (Brucella Medium Base) following the previously described conditions. This process was repeated twice to ensure proper identification of the isolates. After the second liquid culture, 1 ml of each pathobiont and non-pathobiont serotype liquid culture was centrifuged at 6000 x g for 10 min. The supernatant was removed, and the pellet was used to extract bacterial DNA according to the protocol by Centeno-Martinez et al., 2022 [9].

A secondary BRD-pathobiont serotype identification step was executed via Sanger sequencing. The extracted DNA of *M. haemolytica* (Mh18, Mh19, Mh27, and Mh70) and *P. multocida* (Pm63, Pm85, and Pm103) pathogenic and non-pathogenic isolates were used to generate 16 S rRNA amplicons using the same PCR protocol and primers mentioned in the 16 S rRNA qPCR step. Each pathogenic and non-pathogenic 16 S rRNA amplicon was sequenced via Sanger sequencing (Eurofins, USA). Multiple sequence alignments were conducted for the *P. multocida* and *M. haemolytica* subspecies. Two *P. multocida* 16 S rRNA reference gene sequences (*P. multocida* strain P1933 and P030653/1) from NCBI were used as references in the Pm subspecies alignment. Similarly, two *M. haemolytica* 16 S rRNA reference gene sequences (*M. haemolytica* strain 90826 and 120731) were selected as references for the *M. haemolytica* sequence alignment. The extracted DNA from each of the pathogenic *M. haemolytica* (Mh70, serotype A1, and Mh19, serotype A6) and *P. multocida* (Pm6, serotype A) isolates was employed to construct the qPCR standard curve for quantifying the abundance of the *P. multocida* and *M. haemolytica* pathogenic serotypes. Additionally, the extracted DNA of the non-pathogenic serotypes was included in the process as negative controls for both PCR and qPCR. For the *P. multocida* PCR assay, a 50 µl volume was prepared, comprising 32.5 µl of iTaq™ Universal Probes Supermix (BioRad, CA, USA), 12.5 µl of Primer/Probe mix (Integrated DNA Technologies IDT, Coralville, Iowa, USA) listed in (see Additional File: Table S1) with a primer concentration of 0.5 µmol/µl and probe concentration of 0.3 µmol/µl [76], 3 µl of PCR-grade water, and 2 µl of nucleic acid template. The PCR cycling conditions followed those reported in Wang et al. (2023) [76], with an annealing temperature of 59 °C. PCR for *M. haemolytica* A1 and A6 was conducted in a 50 µl reaction, consisting of 32.5 µl of LightCycler 480 SYBR Green I Master (Thermo Fisher Scientific, PA, USA), 12.5 µl of both primers at a concentration of 0.8 µM, 3 µl of PCR-grade water, and 2 µl of nucleic acid template. PCR cycling conditions were carried out following Klima et al. (2017) [77].

Negative controls, including PCR-grade water and non-pathogenic serotypes of *P. multocida* and *M. haemolytica*, were included. PCR amplification was verified through gel-electrophoresis. Cleanup and dilution of *P. multocida* and *M. haemolytica* amplicons, as well as the generation of the standard curve equation, were performed as described in Centeno-Martinez et al. (2022) [9]. The qPCR technical triplicate for pathobionts was carried out in a 20 µl volume, containing 13 µl of iTaq™ Universal Probes Supermix (BioRad, CA, USA) for *P. multocida* or LightCycler 480 SYBR Green I Master (Thermo Fisher Scientific, PA, USA) for *M. haemolytica*, followed by the addition of 5 µl of each primer/probe, as described above, and 2 µl of nucleic acid template. The qPCR cycling conditions for each of the pathogen serotypes were performed as previously described. To assess the specificity of the BRD-pathobiont serotype primers, samples from IN (*n* = 83) and NY (*n* = 76) were subjected to the analysis. Quantification of the pathogenic serotype abundance was carried out in triplicate, as defined in Centeno-Martinez et al. (2022) [9]. The prevalence of each of the pathogenic serotypes was determined using the standard curve LOD (see Additional File: Table S4).

### Statistical analysis for 16 S rRNA gene sequencing and abundance of BRD-pathobionts

Prior to statistical testing, normality of residuals and homogeneity of variance were checked using the afex package [78]. Normality of raw and log-transformed data was checked using the Shapiro-Wilk normality test. Unfortunately, the alpha diversity metrics and the abundance of the BRD-pathobionts failed (*P* > 0.05) the normality of the residual assumption after transformation. Thus, non-parametric methods were applied to evaluate the effect of disease status and collection site (farm) on the microbial alpha diversity and abundance of BRD-pathobionts and 16 S rRNA gene.

To identify the overall effect of disease status (BRD-affected compared to apparently healthy), a Mann-Whitney test, using the function ‘mwu’ from the package sjstats in R [79], was applied in the alpha diversity and the abundance of BRD-pathobionts and 16 S rRNA gene. Then, samples were separated into each farm (CA, IN, NY, TX) to identify any differences in the alpha diversity, abundance of BRD-pathobionts, and 16 S rRNA gene within each farm using a Mann-Whitney test. Additionally, a chi-squared test was performed to identify significant difference in the BRD-pathobiont prevalence within each farm. Similar statistical tests were applied to determine if the abundance and prevalence of the *P. multocida* and *M. haemolytica* pathogenic serotypes were different between the two groups. Additionally, to identify the overall effect among the farms in the alpha diversity and abundance of BRD-pathobionts and 16 S rRNA gene, a Kruskal-Wallis test was applied using the function ‘kruskal.test’ in R. *P* values were adjusted using the Benjamini-Hochberg. Statistical significance was specified as *P* < 0.05.

## Electronic supplementary material

Below is the link to the electronic supplementary material.

Supplementary Material 1

## Data Availability

Nasal swabs sequences divided by Farm (CA, IN, NY and TX) and DNA extraction negative control sequences were deposited in the NCBI sequence read archive (SRA) database as following: CA samples: Bioproject PRJNA1075687, IN samples: Bioproject PRJNA1075699, NY samples: Bioproject PRJNA1075708, and TX samples: BioprojectPRJNA1075746. DNA negative controls are found in the Bioproject PRJNA1075768, Biosamples SAMN39924150- SAMN39924172. Additional files used in data analysis for this study are available at https://github.com/EuniceCenteno/BigBRDProject for reference and reproducibility.

## References

[1] Edward AJ. Respiratory disease in feedlot cattle in the central USA. Bov Pr. 1996;30:5–7.

[2] Snowder GD, Van Vleck LD, Cundiff LV, Bennett GL. Bovine respiratory disease in feedlot cattle: Environmental, genetic, and economic factors. J Anim Sci. 2006;84:1999–2008. 10.2527/jas.2006-046 16864858 10.2527/jas.2006-046

[3] Casella E, Cantor MC, Silvestri S, Renaud DL, Costa JHC. (2022). Cost-aware inference of bovine respiratory disease in calves using precision livestock technology. Proceedings– 18th Annual International Conference on Distributed Computing in Sensor Systems, DCOSS 2022, 109–116. 10.1109/DCOSS54816.2022.00031

[4] USDA. Dairy 2014 dairy cattle management practices in the United States. Natl Anim Heal Monit Syst. 2016; February:135. http://www.aphis.usda.gov/nahms

[5] USDA. Death loss in U. S. Cattle and calves due to predator and nonpredator causes. USDA–APHIS–VS–CEAH: December; 2017.

[6] Cortese VS. Neonatal immunology. Vet Clin North Am Food Anim Pract. 2009, 25:221–7. 10.1016/J.CVFA.2008.10.003 10.1016/j.cvfa.2008.10.003 PMC7126137 19174291

[7] Ferraro S, Fecteau G, Dubuc J, Francoz D, Rousseau M, Roy JP, Buczinski S. Scoping review on clinical definition of bovine respiratory disease complex and related clinical signs in dairy cows. J Dairy Sci. 2021;104:7095–108. 10.3168/jds.2020-19471 33741167 10.3168/jds.2020-19471

[8] White BJ, Renter DG. Bayesian estimation of the performance of using clinical observations and harvest lung lesions for diagnosing bovine respiratory disease in post-weaned beef calves. J Vet Diagn Invest. 2009;21:446–53. 10.1177/104063870902100405 19564492 10.1177/104063870902100405

[9] Centeno-Martinez RE, Glidden N, Mohan S, Davidson JL, Fernández-Juricic E, Boerman JP, Schoonmaker J, Pillai D, Koziol J, Ault A, Verma MS, Johnson TA. Identification of bovine respiratory disease through the nasal microbiome. Anim Microbiome. 2022;4:1–18. 10.1186/s42523-022-00167-y 35193707 10.1186/s42523-022-00167-y PMC8862248

[10] Cirone F, Padalino B, Tullio D, Capozza P, Surdo M, Lo, Lanave G, Pratelli A. Prevalence of pathogens related to bovine respiratory disease before and after transportation in beef steers: preliminary results. Anim. 2019;9. 10.3390/ANI9121093 10.3390/ani9121093 PMC6940923 31817737

[11] Holman DB, McAllister TA, Topp E, Wright ADG, Alexander TW. The nasopharyngeal microbiota of feedlot cattle that develop bovine respiratory disease. Vet Microbiol. 2015;180:90–5. 10.1016/j.vetmic.2015.07.031 26249828 10.1016/j.vetmic.2015.07.031

[12] McMullen C, Alexander TW, Orsel K, Timsit E. Progression of nasopharyngeal and tracheal bacterial microbiotas of feedlot cattle during development of bovine respiratory disease. Vet Microbiol. 2020;248:108826. 10.1016/j.vetmic.2020.108826 32891954 10.1016/j.vetmic.2020.108826

[13] Pansri P, Katholm J, Krogh KM, Aagaard AK, Schmidt LMB, Kudirkiene E, Larsen LE, Olsen JE. Evaluation of novel multiplex qPCR assays for diagnosis of pathogens associated with the bovine respiratory disease complex. Vet J. 2020;256105425. 10.1016/j.tvjl.2020.105425 10.1016/j.tvjl.2020.105425 PMC7110767 32113583

[14] Pratelli A, Cirone F, Capozza P, Trotta A, Corrente M, Balestrieri A, Buonavoglia C. Bovine respiratory disease in beef calves supported long transport stress: an epidemiological study and strategies for control and prevention. Res Vet Sci. 2021;135:450–5. 10.1016/J.RVSC.2020.11.002 33203584 10.1016/j.rvsc.2020.11.002

[15] Gaudino M, Nagamine B, Ducatez MF, Meyer G. Understanding the mechanisms of viral and bacterial coinfections in bovine respiratory disease: a comprehensive literature review of experimental evidence. Vet Res. 2022;53:70. 10.1186/s13567-022-01086-1 36068558 10.1186/s13567-022-01086-1 PMC9449274

[16] Klima CL, Zaheer R, Cook SR, Booker CW, Hendrick S, Alexander TW, McAllister TA. Pathogens of bovine respiratory disease in north American feedlots conferring multidrug resistance via integrative conjugative elements. J Clin Microbiol. 2014;52:438–48. 10.1128/JCM.02485-13 24478472 10.1128/JCM.02485-13 PMC3911356

[17] Mosier D. Review of BRD pathogenesis: the old and the new. Anim Heal Res Rev. 2014;24:166–9. 10.1017/S1466252314000176 10.1017/S1466252314000176 25351390

[18] Zeineldin M, Lowe J, Aldridge B. Contribution of the mucosal microbiota to bovine respiratory health. Trends Microbiol. 2019;27:753–70. 10.1016/j.tim.2019.04.005 31104970 10.1016/j.tim.2019.04.005

[19] Rice JA, Carrasco-Medina L, Hodgins DC, Shewen PE. *Mannheimia haemolytica* and bovine respiratory disease. Anim Health Res Reviews. 2008;8:117–28. 10.1017/S1466252307001375 10.1017/S1466252307001375 18218156

[20] Zecchinon L, Fett T, Desmecht D. How *Mannheimia haemolytica* defeats host defence through a kiss of death mechanism. Vet Res. 2005;36:133–56. 10.1051/vetres:2004065 15720968 10.1051/vetres:2004065

[21] Dabo SM, Taylor JD, Confer AW. *Pasteurella multocida* and bovine respiratory disease. Anim Heal Res Rev. 2008;8:129–50. 10.1017/S1466252307001399 10.1017/S1466252307001399 18218157

[22] Kumar AA, Shivachandra SB, Biswas A, Singh VP, Singh VP, Srivastava SK. Prevalent serotypes of *Pasteurella multocida* isolated from different animal and avian species in India. Vet Res Commun. 2004;28:657–67. 10.1023/B:VERC.0000045959.36513.e9 15609866 10.1023/b:verc.0000045959.36513.e9

[23] Siddaramappa S, Challacombe JF, Duncan AJ, Gillaspy AF, Carson M, Gipson J, Orvis J, Zaitshik J, Barnes G, Bruce D, Chertkov O, Detter JC, Han CS, Tapia R, Thompson LS, Dyer DW, Inzana TJ. Horizontal gene transfer in *Histophilus somni* and its role in the evolution of pathogenic strain 2336, as determined by comparative genomic analyses. BMC Genomics. 2011;12:1–20. 10.1186/1471-2164-12-570 10.1186/1471-2164-12-570 PMC3339403 22111657

[24] Zekarias B, Mattoo S, Worby C, Lehmann J, Rosenbusch RF, Corbeil LB. *Histophilus somni* IbpA DR2/Fic in virulence and immunoprotection at the natural host alveolar epithelial barrier. Infect Immun. 2010;78:1850–8. 10.1128/IAI.01277-09 20176790 10.1128/IAI.01277-09 PMC2863524

[25] Gupta VK, Paul S, Dutta C. Geography, ethnicity or subsistence-specific variations in human microbiome composition and diversity. Front Microbiol. 2017;8:1162. 10.3389/fmicb.2017.01162 28690602 10.3389/fmicb.2017.01162 PMC5481955

[26] Karle BM, Maier GU, Love WJ, Dubrovsky SA, Williams DR, Anderson RJ, Van Eenennaam AL, Lehenbauer TW, Aly SS. Regional management practices and prevalence of bovine respiratory disease in California’s preweaned dairy calves. J Dairy Sci. 2019;102(8):7583–96. 10.3168/jds.2018-14775 30527977 10.3168/jds.2018-14775

[27] McMullen C, Alexander TW, Léguillette R, Workentine M, Timsit E. Topography of the respiratory tract bacterial microbiota in cattle. Microbiome. 2020;8. 10.1186/s40168-020-00869-y 10.1186/s40168-020-00869-y PMC7288481 32522285

[28] Welter DK, Ruaud A, Henseler ZM, Jong HN, De, Groot P, van Michaux C, Gormezano J, Waters L, Youngblut JL, Ley ND. Free-living, psychrotrophic bacteria of the genus *Psychrobacter* are descendants of pathobionts. mSystems. 2021;6:2. 10.1128/MSYSTEMS.00258-21 10.1128/mSystems.00258-21 PMC8546975 33850039

[29] Chai J, Liu X, Usdrowski H, Deng F, Li Y, Zhao J. Geography, niches, and transportation influence bovine respiratory microbiome and health. Front Cell Infect Microbiol. 2022;12:961644. 10.3389/FCIMB.2022.961644 36171758 10.3389/fcimb.2022.961644 PMC9510686

[30] Nicola I, Cerutti F, Grego E, Bertone I, Gianella P, D’Angelo A, Peletto S, Bellino C. Characterization of the upper and lower respiratory tract microbiota in piedmontese calves. Microbiome. 2017;5:152. 10.1186/S40168-017-0372-5 29157308 10.1186/s40168-017-0372-5 PMC5697440

[31] Lima SF, Teixeira AGV, Higgins CH, Lima FS, Bicalho RC. The upper respiratory tract microbiome and its potential role in bovine respiratory disease and otitis media. Sci Rep. 2016;6:1–12. 10.1038/srep29050 27363739 10.1038/srep29050 PMC4929571

[32] Packiavathy IASV, Kannappan A, Thiyagarajan S, Srinivasan R, Jeyapragash D, Paul JBJ, Velmurugan P, Ravi AV. AHL-lactonase producing *Psychrobacter* sp. from palk bay sediment mitigates Quorum sensing-mediated virulence production in gram negative bacterial pathogens. Front Microbiol. 2021;12:634593. 10.3389/fmicb.2021.634593 33935995 10.3389/fmicb.2021.634593 PMC8079732

[33] Zhang Z, Zhang C, Zhong Y, Yang S, Deng F, Li Y, Chai J. The spatial dissimilarities and connections of the microbiota in the upper and lower respiratory tract of beef cattle. Front Cell Infect Microbiol. 2023;13:1269726. 10.3389/FCIMB.2023.1269726 38029262 10.3389/fcimb.2023.1269726 PMC10660669

[34] McDaneld TG, Kuehn LA, Keele JW. Evaluating the microbiome of two sampling locations in the nasal cavity of cattle with bovine respiratory disease complex (BRDC) 1. J Anim Sci. 2018;96:1281–7. 10.1093/JAS/SKY032 29659872 10.1093/jas/sky032 PMC6140963

[35] Zeineldin M, Lowe J, De Godoy M, Maradiaga N, Ramirez C, Ghanem M, Abd El-Raof Y, Aldridge B. Disparity in the nasopharyngeal microbiota between healthy cattle on feed, at entry processing and with respiratory disease. Vet Microbiol. 2017;208:30–7. 10.1016/j.vetmic.2017.07.006 28888646 10.1016/j.vetmic.2017.07.006

[36] McMullen C, Orsel K, Alexander TW, van der Meer F, Plastow G, Timsit E. Comparison of the nasopharyngeal bacterial microbiota of beef calves raised without the use of antimicrobials between healthy calves and those diagnosed with bovine respiratory disease. Vet Microbiol. 2019;231:56–62. 10.1016/j.vetmic.2019.02.030 30955824 10.1016/j.vetmic.2019.02.030

[37] Timsit E, Workentine M, van der Meer F, Alexander T. Distinct bacterial metacommunities inhabit the upper and lower respiratory tracts of healthy feedlot cattle and those diagnosed with bronchopneumonia. Vet Microbiol. 2018;221:105–13. 10.1016/j.vetmic.2018.06.007 29981695 10.1016/j.vetmic.2018.06.007

[38] Cardinale BJ, Duffy JE, Gonzalez A, Hooper DU, Perrings C, Venail P, Narwani A, MacE GM, Tilman D, Wardle DA, Kinzig AP, Daily GC, Loreau M, Grace JB, Larigauderie A, Srivastava DS, Naeem S. Biodiversity loss and its impact on humanity. Nature. 2012;486:59–67. 10.1038/nature11148 22678280 10.1038/nature11148

[39] Knapp S, Winter M, Klotz S. Increasing species richness but decreasing phylogenetic richness and divergence over a 320-year period of urbanization. J Appl Ecol. 2017;54:1152–60. 10.1111/1365-2664.12826

[40] Bissett A, Brown MV, Siciliano SD, Thrall PH. Microbial community responses to anthropogenically induced environmental change: Towards a systems approach. Ecol Lett. 2013, 16 SUPPL.1:128–39. 10.1111/ele.12109 10.1111/ele.12109 23679012

[41] Griffin D, Chengappa MM, Kuszak J, McVey DS. Bacterial pathogens of the bovine respiratory disease complex. Veterinary Clin North Am - Food Anim Pract. 2010;26:381–94. 10.1016/j.cvfa.2010.04.004 10.1016/j.cvfa.2010.04.004 20619191

[42] Goto Y, Fukunari K, Suzuki T, Multiplex. RT-qPCR application in early detection of bovine respiratory disease in healthy calves. Viruses. 2023;15:669. 10.3390/V15030669 36992378 10.3390/v15030669 PMC10057971

[43] Thomas AC, Bailey M, Lee MRF, Mead A, Morales-Aza B, Reynolds R, Vipond B, Finn A, Eisler MC. Insights into *Pasteurellaceae* carriage dynamics in the nasal passages of healthy beef calves. Sci Rep. 2019;9:1–14. 10.1038/s41598-019-48007-5 31420565 10.1038/s41598-019-48007-5 PMC6697682

[44] Valeris-Chacin R, Powledge S, McAtee T, Morley PS, Richeson J. *Mycoplasma bovis* is associated with *Mannheimia haemolytica* during acute bovine respiratory disease in feedlot cattle. Front Microbiol. 2022;13:946792. 10.3389/FMICB.2022.946792 35979489 10.3389/fmicb.2022.946792 PMC9376970

[45] Wisselink HJ, Cornelissen JBWJ, van der Wal FJ, Kooi EA, Koene MG, Bossers A, Smid B, de Bree FM, Antonis AFG. Evaluation of a multiplex real-time PCR for detection of four bacterial agents commonly associated with bovine respiratory disease in bronchoalveolar lavage fluid. BMC Vet Res. 2017;13. 10.1186/s12917-017-1141-1 10.1186/s12917-017-1141-1 PMC5508684 28705198

[46] Gautam R, Kumar AA, Singh VP, Singh VP, Dutta TK, Shivachandra SB. Specific identification of *Pasteurella multocida* serogroup-A isolates by PCR assay. Res Vet Sci. 2004;76:179–85. 10.1016/j.rvsc.2003.10.005 15046950 10.1016/j.rvsc.2003.10.005

[47] Kudirkiene E, Aagaard AK, Schmidt LMB, Pansri P, Krogh KM, Olsen JE. Occurrence of major and minor pathogens in calves diagnosed with bovine respiratory disease. Vet Microbiol. 2021;259:109135. 10.1016/j.vetmic.2021.109135 34090248 10.1016/j.vetmic.2021.109135

[48] Centeno-Martinez RE, Klopp BN, Koziol J, Boerman JP, Johnson TA. Dynamics of the nasopharyngeal microbiome of apparently healthy calves and those with clinical symptoms of BRD from disease diagnosis to recovery. Front Veterinary Sci. 2023;10:1297158. 10.3389/fvets.2023.1297158 10.3389/fvets.2023.1297158 PMC10687565 38033643

[49] McMullen C, Orsel K, Alexander TW, van der Meer F, Plastow G, Timsit E. Evolution of the nasopharyngeal bacterial microbiota of beef calves from spring processing to 40 days after feedlot arrival. Vet Microbiol. 2018;225:139–48. 10.1016/j.vetmic.2018.09.019 30322526 10.1016/j.vetmic.2018.09.019

[50] He Y, Wu W, Zheng HM, Li P, McDonald D, Sheng HF, Chen MX, Chen ZH, Ji GY, Zheng ZD, Mujagond P, Chen XJ, Rong ZH, Chen P, Lyu LY, Wang X, Wu CB, Yu N, Xu YJ, Yin J, Raes J, Knight R, Ma WJ, Zhou HW. Regional variation limits applications of healthy gut microbiome reference ranges and disease models. Nat Med. 2018;24:1532–5. 10.1038/s41591-018-0164-x 30150716 10.1038/s41591-018-0164-x

[51] Gilbert JA, Quinn RA, Debelius J, Xu ZZ, Morton J, Garg N, Jansson JK, Dorrestein PC, Knight R. Microbiome-wide association studies link dynamic microbial consortia to disease. Nat. 2016;535(7610):94–103. 10.1038/nature18850 10.1038/nature18850 27383984

[52] Fulton RW. Viral diseases of the bovine respiratory tract. Food Anim Pract. 2009;171–91. 10.1016/B978-141603591-6.10042-9

[53] Kirchhoff J, Uhlenbruck S, Goris K, Keil GM, Herrler G. Three viruses of the bovine respiratory disease complex apply different strategies to initiate infection. Veterinary Resesearch. 2014;45:1–12. 10.1186/1297-9716-45-20 10.1186/1297-9716-45-20 PMC3942114 24548739

[54] Hodgins DC, Conlon JA, Shewen PE. Respiratory viruses and bacteria in cattle. Polymicrobial Dis. 2014;213–29. 10.1128/9781555817947.ch12

[55] Kano R, Konishi K, Nakata K, Sano K, Komatsu S, Nomura M, Okuzumi K, Hasegawa A. Isolation of *Candida krusei* from a case of bovine bronchopneumonia in a one-year-old heifer. Vet Rec. 2001;148:636. 10.1136/vr.148.20.636 11394804 10.1136/vr.148.20.636

[56] Abdelfattah EM, Aly SS, Lehenbauer TW, Karle BM. Effects of simplified group housing on behavior, growth performance and health of preweaned dairy calves on a California dairy. J Dairy Sci 2024 S0022-0302(24)00017– 1. 10.3168/jds.2023-23820 10.3168/jds.2023-23820 38246538

[57] Holman DB, Timsit E, Alexander TW. The nasopharyngeal microbiota of feedlot cattle. Sci Rep. 2015;51. 10.1038/srep15557 10.1038/srep15557 PMC4620444 26497574

[58] Kozich JJ, Westcott SL, Baxter NT, Highlander SK, Schloss PD. Development of a dual-index sequencing strategy and curation pipeline for analyzing amplicon sequence data on the MiSeq Illumina sequencing platform. Appl Environ Microbiol. 2013;79:5112–20. 10.1128/AEM.01043-13 23793624 10.1128/AEM.01043-13 PMC3753973

[59] Callahan BJ, McMurdie PJ, Rosen MJ, Han AW, Johnson AJA, Holmes SP. DADA2: high-resolution sample inference from Illumina amplicon data. Nat Methods. 2016;13:581. 10.1038/nmeth.3869 27214047 10.1038/nmeth.3869 PMC4927377

[60] Mandal S, Van Treuren W, White RA, Eggesbø M, Knight R, Peddada SD. Analysis of composition of microbiomes: a novel method for studying microbial composition. Microb Ecol Health Dis. 2015;26. 10.3402/MEHD.V26.27663 10.3402/mehd.v26.27663 PMC4450248 26028277

[61] Lin H, Peddada S, Das. Analysis of compositions of microbiomes with bias correction. Nat Commun. 2020;11:1–11. 10.1038/s41467-020-17041-7 32665548 10.1038/s41467-020-17041-7 PMC7360769

[62] Chao A. Nonparametric estimation of the number of classes in a Population. Scand J Stat. 1984;11:265–70.

[63] DeSantis TZ, Hugenholtz P, Larsen N, Rojas M, Brodie EL, Keller K, Huber T, Dalevi D, Hu P, Andersen GL. Greengenes, a chimera-checked 16S rRNA gene database and workbench compatible with ARB. Appl Environ Microbiol. 2006;72:5069–72. 10.1128/AEM.03006-05 16820507 10.1128/AEM.03006-05 PMC1489311

[64] Faith DP. Conservation evaluation and phylogenetic diversity. Biol Conserv. 1992;61:1–10. 10.1016/0006-3207(92)91201-3

[65] Hagerty SL, Hutchison KE, Lowry CA, Bryan AD. An empirically derived method for measuring human gut microbiome alpha diversity: demonstrated utility in predicting health related outcomes among a human clinical sample. PLoS ONE. 2020;15:3. 10.1371/journal.pone.0229204 10.1371/journal.pone.0229204 PMC7051054 32119675

[66] Pielou EC. The measurement of diversity in different types of biological collections. J Theor Biol. 1966;13 C:131–44. 10.1016/0022-5193(66)90013-0

[67] Whittaker RH. Vegetation of the Siskiyou Mountains, Oregon and California. Ecol Monogr. 1960;30:279–338. 10.2307/1943563

[68] Lozupone C, Knight R. UniFrac: a new phylogenetic method for comparing microbial communities. Appl Environ Microbiol. 2005;71:8228–35. 10.1128/AEM.71.12.8228-8235.2005 16332807 10.1128/AEM.71.12.8228-8235.2005 PMC1317376

[69] Oksanen J, Blanchet FG, Friendly M, Kindt R, Legendre P, Mcglinn D, Minchin PR, O’Hara RB, Simpson GL, Solymos P, Henry M, Stevens H, Szoecs E, Maintainer HW. Package vegan Title Community Ecology Package Version 2.5-6. 2019.

[70] Galkiewicz JP, Kellogg CA. Cross-kingdom amplification using Bacteria-specific primers: complications for studies of coral microbial ecology. Appl Environ Microbiol. 2008;74:7828–31. 10.1128/AEM.01303-08 18931299 10.1128/AEM.01303-08 PMC2607152

[71] Leigh MB, Pellizari VH, Uhlík O, Sutka R, Rodrigues J, Ostrom NE, Zhou J, Tiedje JM. Biphenyl-utilizing bacteria and their functional genes in a pine root zone contaminated with polychlorinated biphenyls (PCBs). ISME J. 2007;1:134–48. 10.1038/ismej.2007.26 18043623 10.1038/ismej.2007.26

[72] Sheets TR. Leveraging of machine learning to evaluate genotypic-phenotypic concordance of *Pasteurella multocida* isolated from bovine respiratory disease cases. Purdue University Graduate School. 2023. https://www.proquest.com/docview/2838439262/. Accessed 13 Dec 2023. 10.1016/j.vetmic.2026.111136 42470920

[73] Wickware CL. Applied bacterial ecology in livestock systems. Purdue Univ Graduate School. 2022. 10.25394/PGS.21402009.V1. Thesis.

[74] Christensen H, Bisgaard M, Menke T, Liman M, Timsit E, Foster G, Olsen JE. Prediction of *Mannheimia haemolytica* serotypes based on whole genomic sequences. Vet Microbiol. 2021;262:109232. 10.1016/j.vetmic.2021.109232 34509701 10.1016/j.vetmic.2021.109232

[75] Christensen H, Sajid SM, Bisgaard M, Magistrali CF, Massacci FR, Liman M, Menke T, Bischoff H, Olsen JE. Prediction of *Pasteurella multocida* serotypes based on whole genomic sequences. Vet Microbiol. 2022;271. 10.1016/J.VETMIC.2022.109492 10.1016/j.vetmic.2022.109492 35714528

[76] Wang H, Xin L, Wu Y, Liu Y, Yao W, Zhang H, Hu Y, Tong R, Zhu L. Construction of a one-step multiplex real-time PCR assay for the detection of serogroups a, B, and E of *Pasteurella multocida* associated with bovine pasteurellosis. Front Vet Sci. 2023;10. 10.3389/fvets.2023.1193162 10.3389/fvets.2023.1193162 PMC10336434 37448584

[77] Klima CL, Zaheer R, Briggs RE, McAllister TA. A multiplex PCR assay for molecular capsular serotyping of *Mannheimia haemolytica* serotypes 1, 2, and 6. J Microbiol Methods. 2017;139:155–60. 10.1016/j.mimet.2017.05.010 28551457 10.1016/j.mimet.2017.05.010

[78] Singmann H, Bolker B, Westfall J, Aust F, Ben-Shachar MS, Højsgaardm S, Fox J, Lawrence MA, Mertens U, Love J, Lenth R, Bojesen Christensen RH. Package afex Title Analysis of Factorial Experiments. 2021.

[79] Lüdecke D, sjstats. Statistical Functions for Regression Models. 2018. 10.5281/ZENODO.1489175

