# Supplementary material for: Nasal pathobiont abundance does not differ between dairy cattle with or without clinical symptoms of bovine respiratory disease
Source: Anim Microbiome. 2025 Feb 18;7:16. doi: 10.1186/s42523-025-00382-3 (PMC11837595; doi:10.1186/s42523-025-00382-3)
Supplement: Supplementary file 1 — Supplementary Material 1 [file 42523_2025_382_MOESM1_ESM.docx]

**ADDITIONAL FILE 1**

**Nasal Pathobiont Abundance Does Not Differ Between Dairy Cattle with or without Clinical Symptoms of Bovine Respiratory Disease**

Ruth Eunice Centeno-Delphia^1^, Erica A. Long^1^, Audrey C. Ellis^1^, Sarah Hofmann^1^, Kara Mosier^1^, Noelmi Ulloa^2^, Johnnie Junior Cheng^1^, Richard Andrews^1^, Jacquelyn P Boerman^1^, Jennifer Koziol^4^, Mohit S Verma^3,5,6^, Timothy A Johnson^1*^.

^1^ Department of Animal Science, Purdue University, West Lafayette, IN, USA

^2^ Escuela Agrícola Panamericana Zamorano, Valle del Yeguare, Honduras

^3^ Department of Agricultural and Biological Engineering, Purdue University, West Lafayette, IN, USA

^4^ School of Veterinary Medicine, Texas Tech University, Amarillo, TX, USA

^5^ Weldon School of Biomedical Engineering, Purdue University, West Lafayette, IN, USA

^6^ Brick Nanotechnology Center, Purdue University, West Lafayette, IN, USA

**Table S1.** List of published primers to target BRD-pathobionts and serotypes.

**Table S2.** Evaluation of the BRD-pathobionts and total bacteria abundance in the cattle nasal cavity via qPCR.

**Table S3.** List of *M. haemolytica* and *P. multocida* pathogenic and non-pathogenic serotypes

**Table S4.** Evaluation of *P. multocida* serotype A and *M. haemolytica* serotype A1 and A6 using qPCR assays**.**

**Table S5.** Alpha diversity metrics in the nasal cavity of cows and calves collected from IN and NY.

**Figure S1.** Relative abundance of BRD-pathobionts predicted by 16S rRNA gene sequencing in the cattle nasal microbiome.

**Figure S2.** Relative abundance of *M. bovis* **(a)** and *P. multocida* **(b)** based on the 16S rRNA abundance and *M. haemolytica* relative abundance based on the four pathobionts **(c).**

**Figure S3.** Total bacteria abundance between states **(a)** and between healthy and BRD-affected samples from NY **(b).**

**Figure S4.** Prevalence of *M. haemolytica* serotype A1 and A6 and *P. multocida* serotype A in samples collected from IN and NY.

**Figure S5.** Bar plot showing relative abundance of the ASVs found in the controls including *Pseudoalteromonas* **(a).** Relative abundance of *Pseudoalteromonas* among all the controls **(b).** Controls included the water samples “water”, mock, and empty swabs (Dairy and extra).

**Figure S6.** Beta diversity between the two NY farms predicted by Bray Curtis **(a)** and Weighted UniFrac **(b).**

**Figure S7.** Prevalence of the BRD-pathobionts identified via 16S rRNA sequencing and quantified via qPCR based on disease status and farm.

**Figure S8.** Correlation between the BDR-pathobionts relative abundance predicted by 16S rRNA and the relative abundance based on 16S rRNA copy number (log_10_) **(a-c)**. Correlation between the BDR-pathobionts relative abundance predicted by 16S rRNA and the relative abundance based on 16S rRNA copy number (log_10_) for each disease status **(d-f).**

**Table S1.** List of published primers to target BRD-pathobionts and serotypes.

| **Target** | **Target** | **Primer name** | **Sequence (5’-3’)** | **Size** | **Ref** |
| --- | --- | --- | --- | --- | --- |
|  | **gene** |  |  | **(bp)** |  |
| *M. haemolytica* | *sod*A | Mh-SGF | ﻿AGCAGCGACTACTCGTGTTGGTTCAG | 26 | 1 |
|  |  | Mh-SGR | *﻿*AAGACTAAAATCGGATAGCCTGAAACGCCTG | 31 |  |
|  |  | ﻿Mh-BV1P^Ψ^ | *﻿*TTCAACCGCTAACCAGGACAACCCAC | 26 |  |
| *P. multocida* | 16S rRNA | Pm-TMF | ﻿CGCAGGCAATGAATTCTCTTC | 21 | 2 |
|  |  | Pm-TMR | ﻿GGCGCTCTTCAGCTGTTTTT | 20 |  |
|  |  | ﻿Pm-TMP^Ψ^ | ﻿ACTGCACCAACAAATGCTTGCTGAGTTAGC | 30 |  |
| *H. somni* | 16S rRNA | Hs-TMF | ﻿AGGAAGGCGATTAGTTTAAGAGATTAATT | 29 | 2 |
|  |  | Hs-TMR | ﻿TCACACCTCACTTAAGTCACCACCT | 25 |  |
|  |  | ﻿Hs-TMP^Ψ^ | ﻿ATTGACGATAATCACAGAAGAAGCACCGGC | 30 |  |
| *M. bovis* | *opp*D | ﻿PMB996-F | ﻿TCAAGGAACCCCACCAGAT | 19 | 3 |
|  |  | ﻿PMB1066-R | ﻿AGGCAAAGTCATTTCTAGGTGCAA | 24 |  |
|  |  | ﻿Mbovis1016^Ψ^ | ﻿TGGCAAACTTACCTATCGGTGACCCT | 26 |  |
| *M. haemolytica* A1 | *HyP* | HyP_F | ﻿﻿CATTTCCTTAGGTTCAGC | 306 | 4 |
|  |  | HyP_R | ﻿﻿CAAGTCATCGTAATGCCT |  |  |
| *M. haemolytica* A6 | *TupA* | ﻿TupA_F | *﻿*﻿TGAGAATTTCGACAGCACT | 78 | 4 |
|  |  | TupA_R | ﻿﻿ACCTTGGCATATCGTACC |  |  |
| *P. multocida* A | HyaD | HyaD_F | ﻿CAGTTTCTCTGGATTGGCGC | 100 | 5 |
|  |  | HyaD_R | ﻿AAAGCAACATTACCCGCCG |  |  |
|  |  | HyaD-Probe* | ﻿FAM-CTCCGCTTATCCGATTCGCCTTTCC-BHQ1 |  |  |
| ^1^Guenther et al., 2008, ^2^ Mahony et al., 2007, ^3^ Sachse et al., 2010, ^4^ Klima et al., 2017, *^5^* Wang et al., 2023 | | | | | |
| ﻿^Ψ^ Probe fluorophore and double quencher: 5′ 6-FAM/ZEN/3′ IBFQ. * Probe fluorophore | | | | | |

**Table S2.** Evaluation of the BRD-pathobionts and total bacteria abundance in the cattle nasal cavity via qPCR.

| **qPCR assay** |  | ***Pasteurella multocida*** | ***Histophilus somni*** | ***Mannheimia haemolytica*** | ***Mycoplasma bovis*** | **16S rRNA gene** |
| --- | --- | --- | --- | --- | --- | --- |
| Standard equation | Slope | -0.313 | -0.270 | -0.316 | -0.297 | -0.324 |
|  | Intercept | 11.784 | 11.182 | 11.38 | 11.172 | 11.699 |
|  | Replicates | 3 | 3 | 3 | 3 | 3 |
| Efficiency (%) |  | 107.28 | 86.74 | 107.96 | 98.40 | 113.11 |
| Dilutions |  | 9 | 9 | 9 | 9 | 9 |
| LOD^Ψ^ |  | 32.863 | 37.385 | 31.76 | 32.51 | 30.52 |
| ^Ψ^ Limit of Detection, Last Cq value in Standard Curve (10^0^) | | | | | | |

**Table S3.** List of *M. haemolytica* and *P. multocida* pathogenic and non-pathogenic serotypes.

| **Bacteria** | **Isolate ID** | **Serotype** |
| --- | --- | --- |
| *P. multocida* | PM63 | Serotype A |
|  | PM85 | Serotype D |
|  | PM103 | Serotype D |
| *M. haemolytica* | MH70 | Serotype A1 |
|  | MH19 | Serotype A6 |
|  | MH18 | Serotype A2 |
|  | MH25 | Serotype A5 |

**Table S4.** Evaluation of *P. multocida* serotype A and *M. haemolytica* serotype A1 and A6 using qPCR assays.

| **qPCR assay** |  | ***P. multocida* A** | ***M. haemolytica* A1** | ***M. haemolytica.* A6** |
| --- | --- | --- | --- | --- |
| Standard equation | Slope | -0.308 | -0.289 | -0.296 |
|  | Intercept | 11.767 | 10.2698 | 10.588 |
|  | Replicates | 3 | 3 | 3 |
| Efficiency (%) |  | 103.350 | 94.93 | 98.008 |
| Dilutions |  | 11 | 11 | 11 |
| LOD^Ψ^ |  | 36.15 | 34.756 | 34.59 |

^Ψ^: Limit of Detection, Last Cq value in Standard Curve (10^0^)

**Analysis between the nasal microbiome in cows and calves sampled from IN and NY**

Nasal swabs collected from IN and NY farm 2, were taken from some fresh cows and calves (IN, cows = 4 and calves = 86; NY Farm 2, cows = 19 and calves = 10). These numbers differ from the values reported in Table 2 because some of the samples were lost due to rarefaction. A separate statistical analysis was performed to identify any differences in the nasal microbiome alpha diversity between the samples collected from cows compared to calves that could introduce noise/bias in the results obtained. We observed that the richness predicted by Chao1 and Observed ASVs were significantly different between cows and calves only in the samples collected from NY. No other significant effect was observed in the NY and IN samples (Table S5). We then tested if removing the cow samples from the NY group would influence the significant effect detected in the alpha diversity richness (Chao1 and Observed ASVs) between apparently healthy and BRD-affected animals. Results from this test demonstrated that the removal of the cow samples does not impact the significant effect of disease status in the nasal microbial richness.

**Table S5.** Alpha diversity metrics in the nasal cavity of cows and calves collected from IN and NY.

| **Farm** | **Groups** | **Alpha** | **Mean.Rank** | **Mann.**  **WhitneyU** | **WilcoxonW** | **Z** | **EffectSize** | **p** | **p.adj** |
| --- | --- | --- | --- | --- | --- | --- | --- | --- | --- |
| NY | Calf vs Cow | Pielou_e | 48.78 vs 52.47 | 3854 | 694 | -0.51 | 0.051 | 0.612 | 0.612 |
|  |  | Chao1 | 54.20 vs 29.97 | 4281.5 | 1121.5 | 3.33 | 0.337 | 0.001 | 0.005 |
|  |  | Observed ASVs | 53.68 vs 32.11 | 4241 | 1081 | 2.97 | 0.300 | 0.003 | 0.008 |
|  |  | Faith PD | 52.43 vs 37.32 | 4142 | 982 | 2.08 | 0.210 | 0.037 | 0.062 |
|  |  | Shannon | 50.73 vs 44.37 | 4008 | 848 | 0.88 | 0.089 | 0.381 | 0.476 |
| IN | Calf vs Cow | Pielou_e | 44.44 vs 68.25 | 3822 | 81 | -1.78 | 0.188 | 0.075 | 0.375 |
|  |  | Chao1 | 45.80 vs 39.00 | 3939 | 198 | 0.51 | 0.054 | 0.611 | 0.652 |
|  |  | Observed ASVs | 45.80 vs 39.00 | 3939 | 198 | 0.51 | 0.054 | 0.611 | 0.652 |
|  |  | Faith PD | 45.77 vs 39.75 | 3936 | 195 | 0.45 | 0.047 | 0.652 | 0.652 |
|  |  | Shannon | 44.91 vs 58.25 | 3862 | 121 | -1.00 | 0.105 | 0.318 | 0.652 |


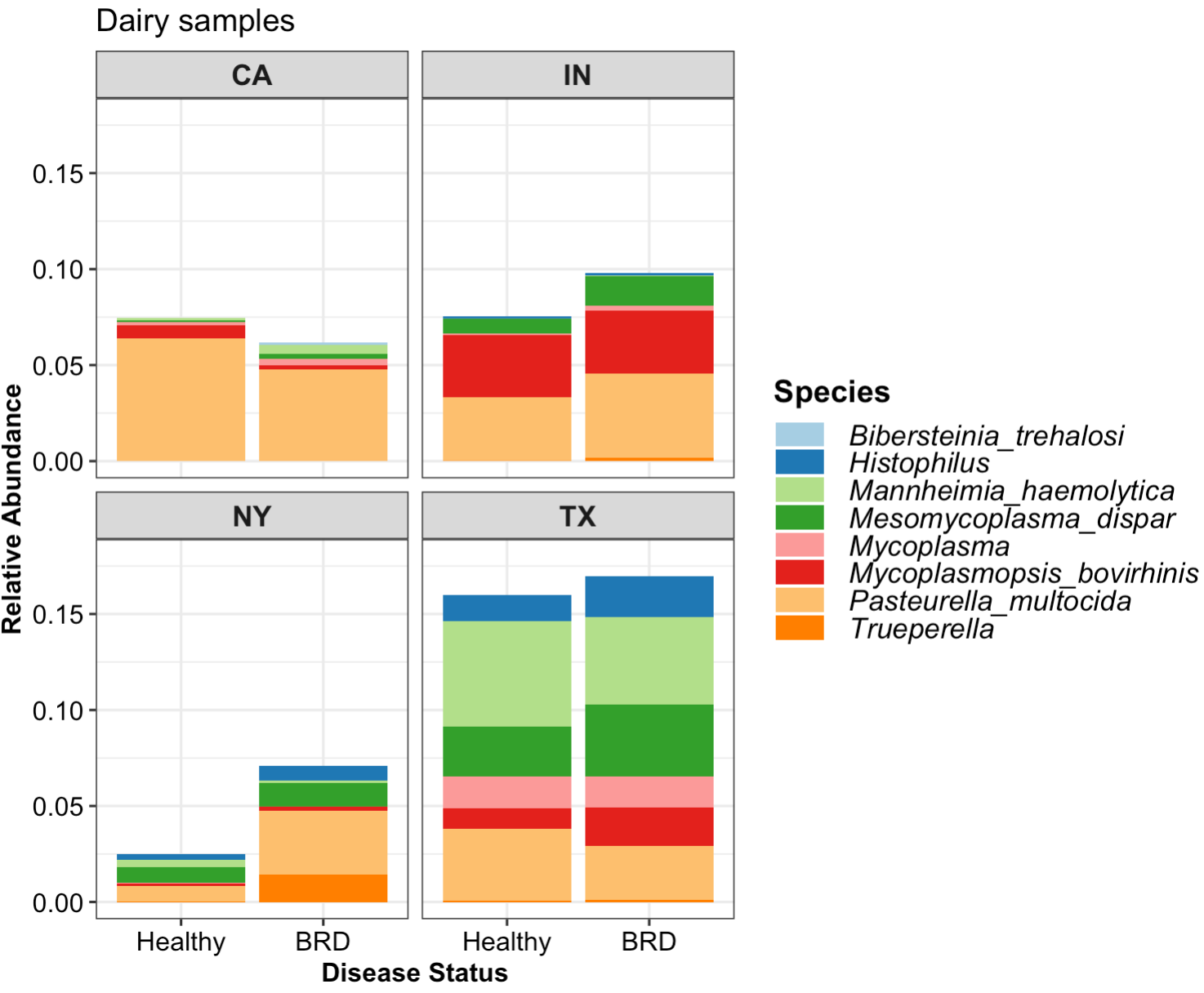


**Figure S1.** Relative abundance of BRD-pathobionts predicted by 16S rRNA gene sequencing in the cattle nasal microbiome.

**
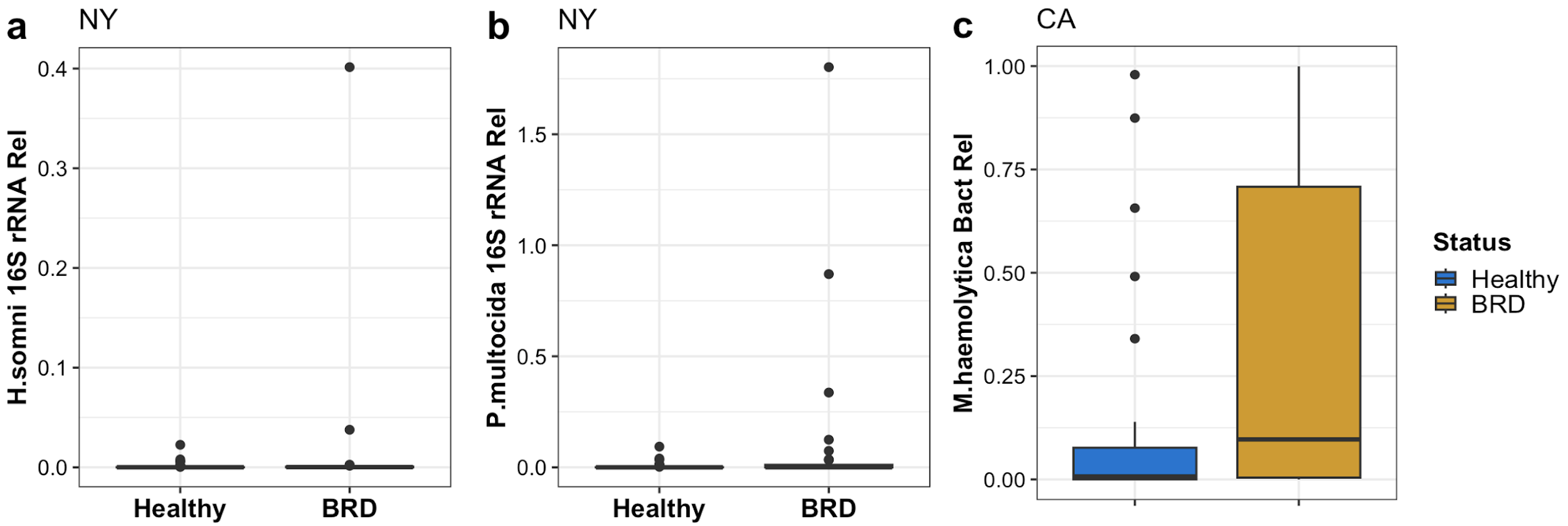
**

**Figure S2.** Abundance of *M. bovis* **(a)** and *P. multocida* **(b)** relative to 16S rRNA abundance and *M. haemolytica* abundance relative to the summed abundance of the four pathobionts **(c).**


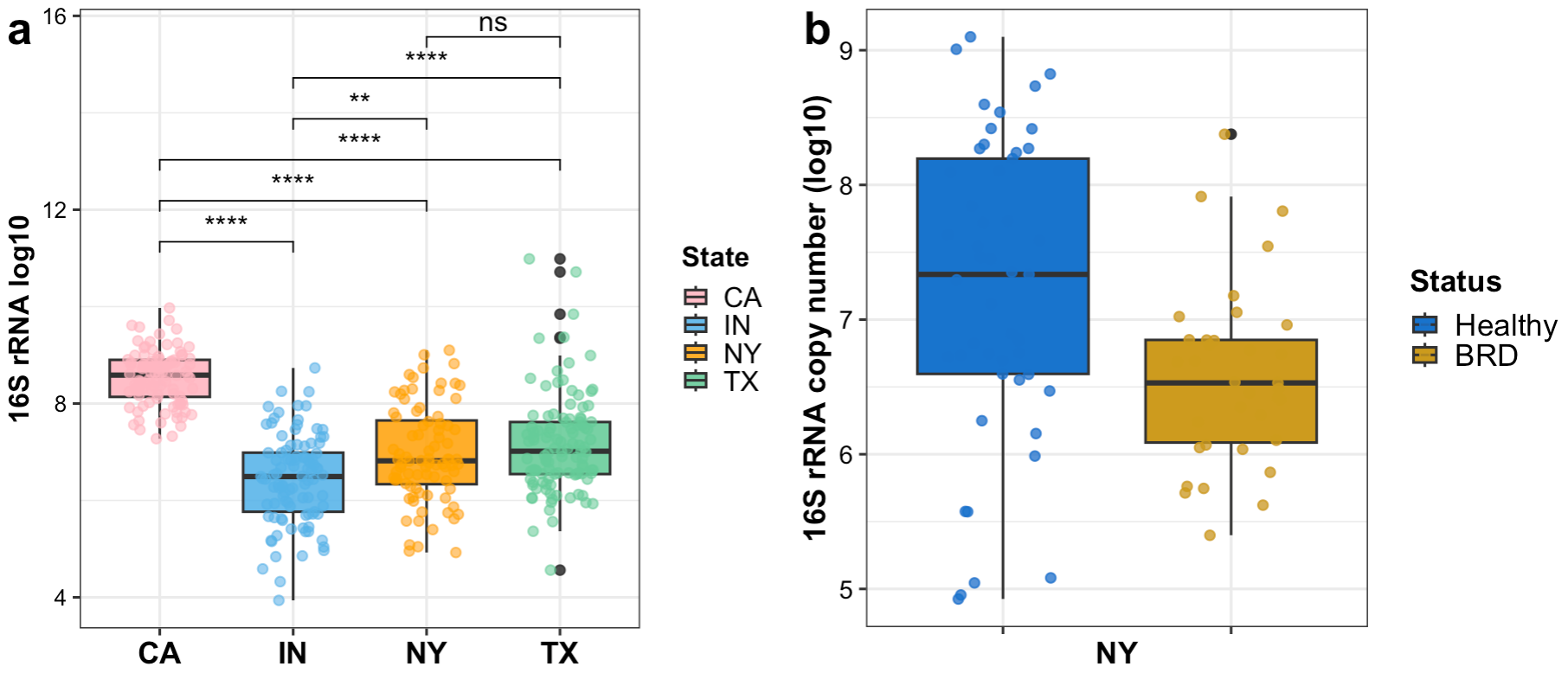


**Figure S3.** Total bacteria abundance between states **(a)** and between healthy and BRD-affected samples from NY **(b).**


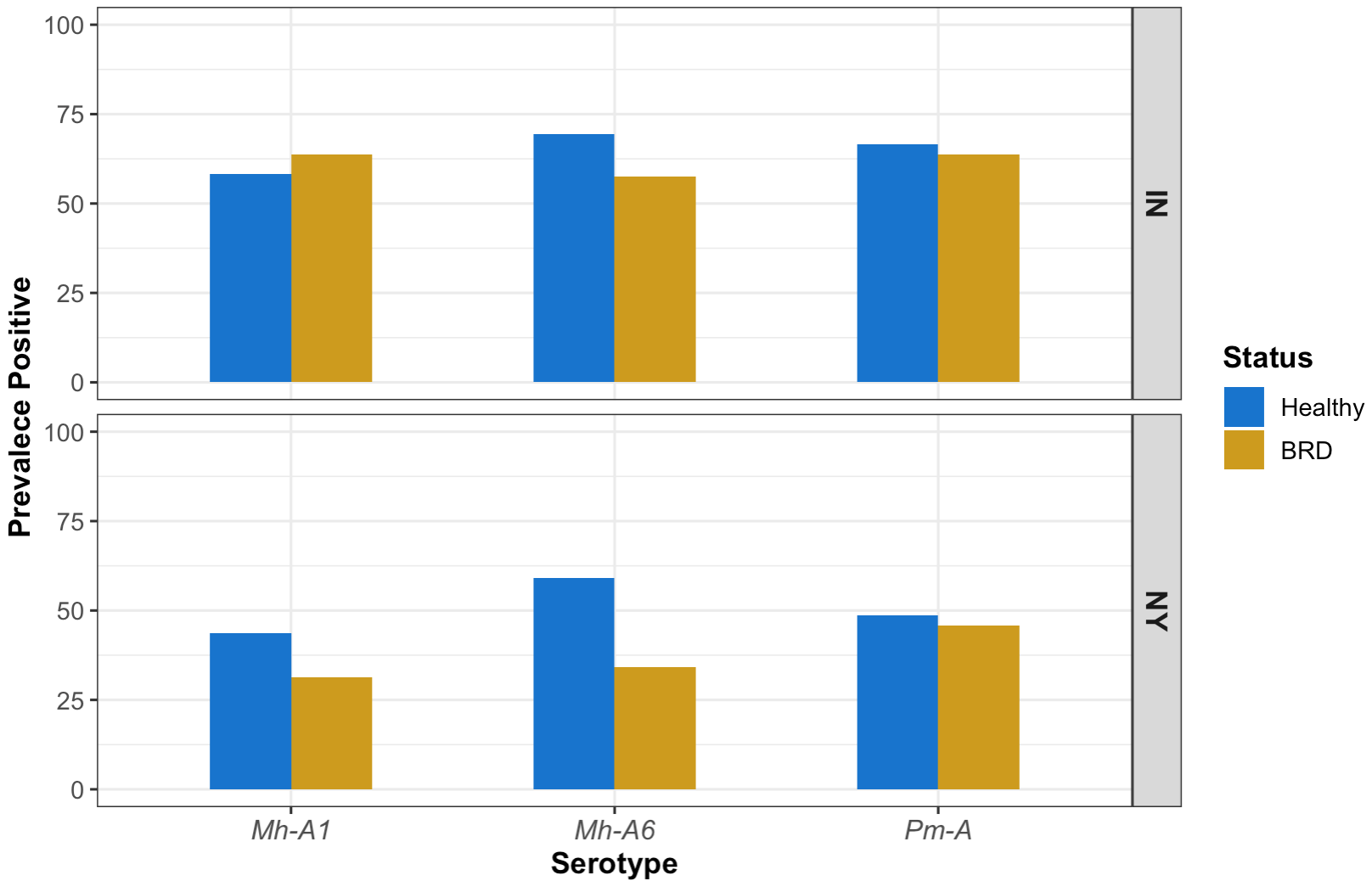


**Figure S4.** Prevalence of *M. haemolytica* serotype A1 and A6 and *P. multocida* serotype A in samples collected from IN and NY.


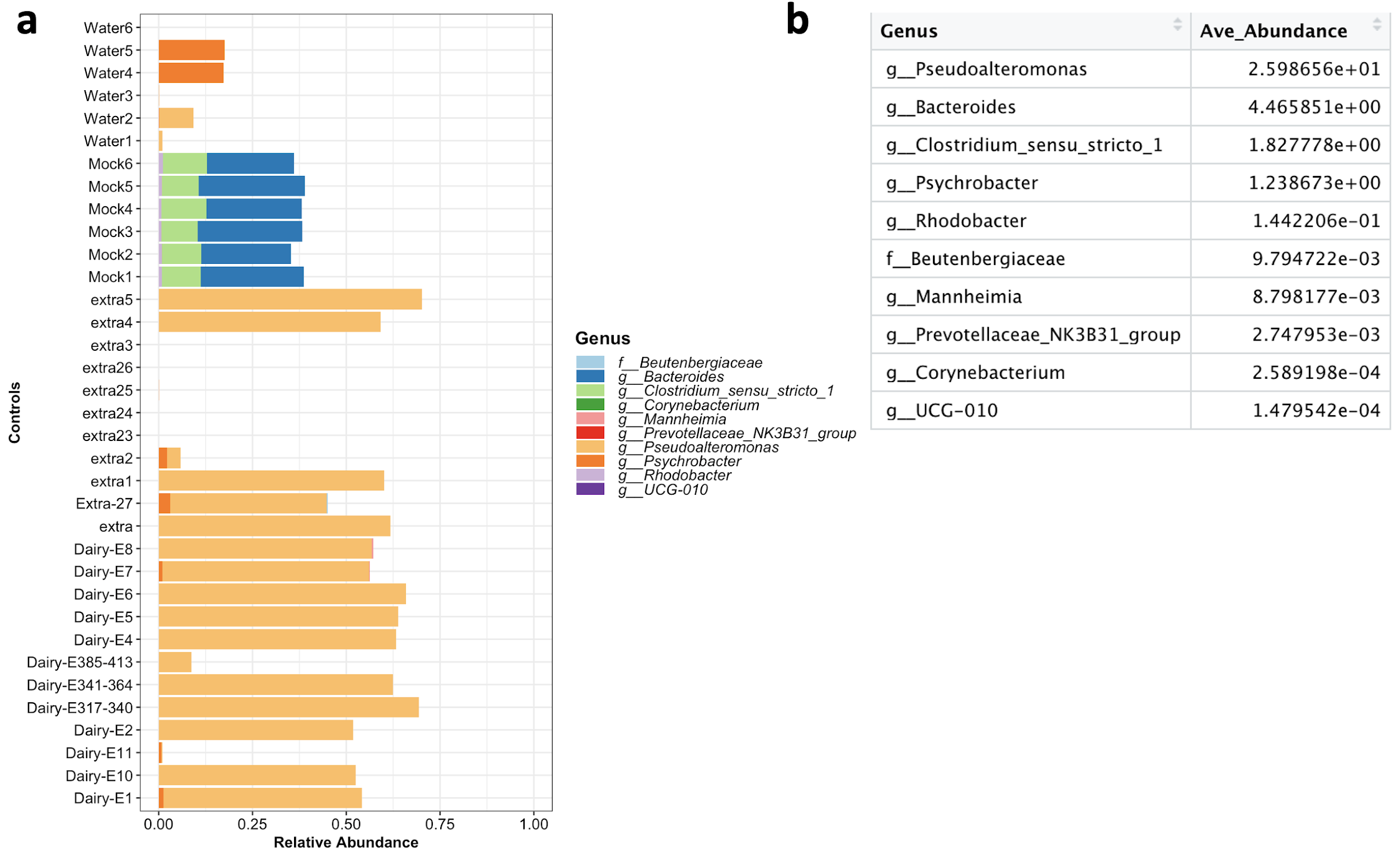


**Figure S5.** Bar plot showing relative abundance of the ASVs found in the controls including *Pseudoalteromonas* **(a).** Relative abundance of *Pseudoalteromonas* among all the controls **(b).** Controls included the water samples “water”, mock, and empty swabs (Dairy and extra).

**Rationale to combine the two NY farms**

The two farms in NY were less than 10 miles apart and therefore the animals were subject to more similar environmental conditions than animals from the other farms. Additionally, we conducted a beta diversity analysis to compare the nasal microbiomes of the two NY farms using Bray-Curtis dissimilarity and Weighted UniFrac. The analysis revealed that while the beta diversity of the communities was significantly different, the effect sizes were low: Bray-Curtis dissimilarity (R² = 0.08248, P = 0.001) and Weighted UniFrac (R² = 0.08558, P = 0.001). The two NY farms were more similar to each other than to any other farm (Fig. S6 a-b) and mostly overlap with each other. The community similarity between these farms maybe due to their geographical proximity, but we cannot prove that. It was also beneficial to combine these farms together to maintain similar sample sizes between states. For these reasons, we decided to combine the two farms into a single group.


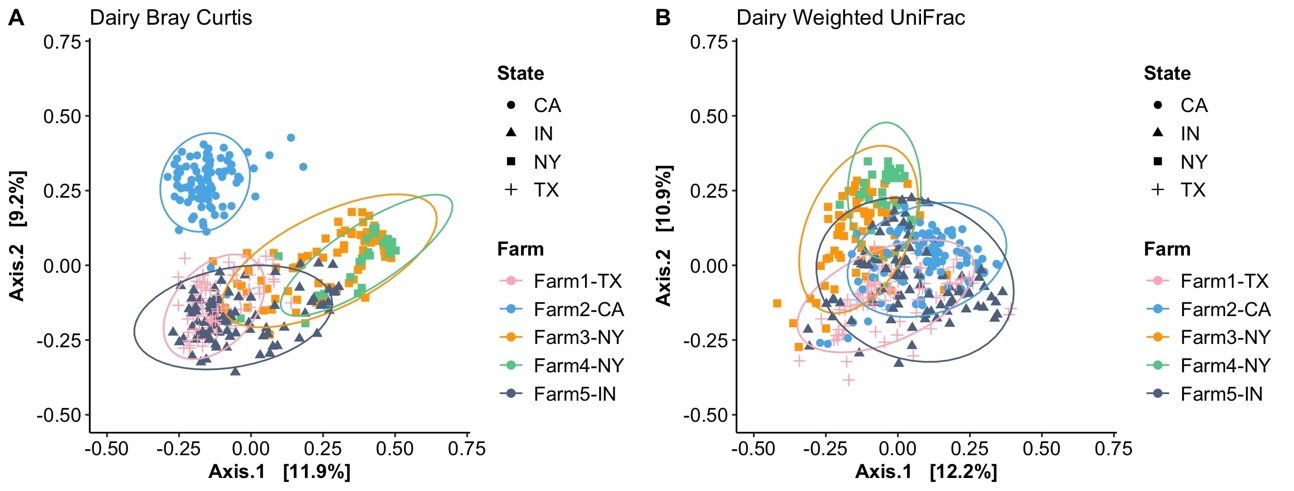


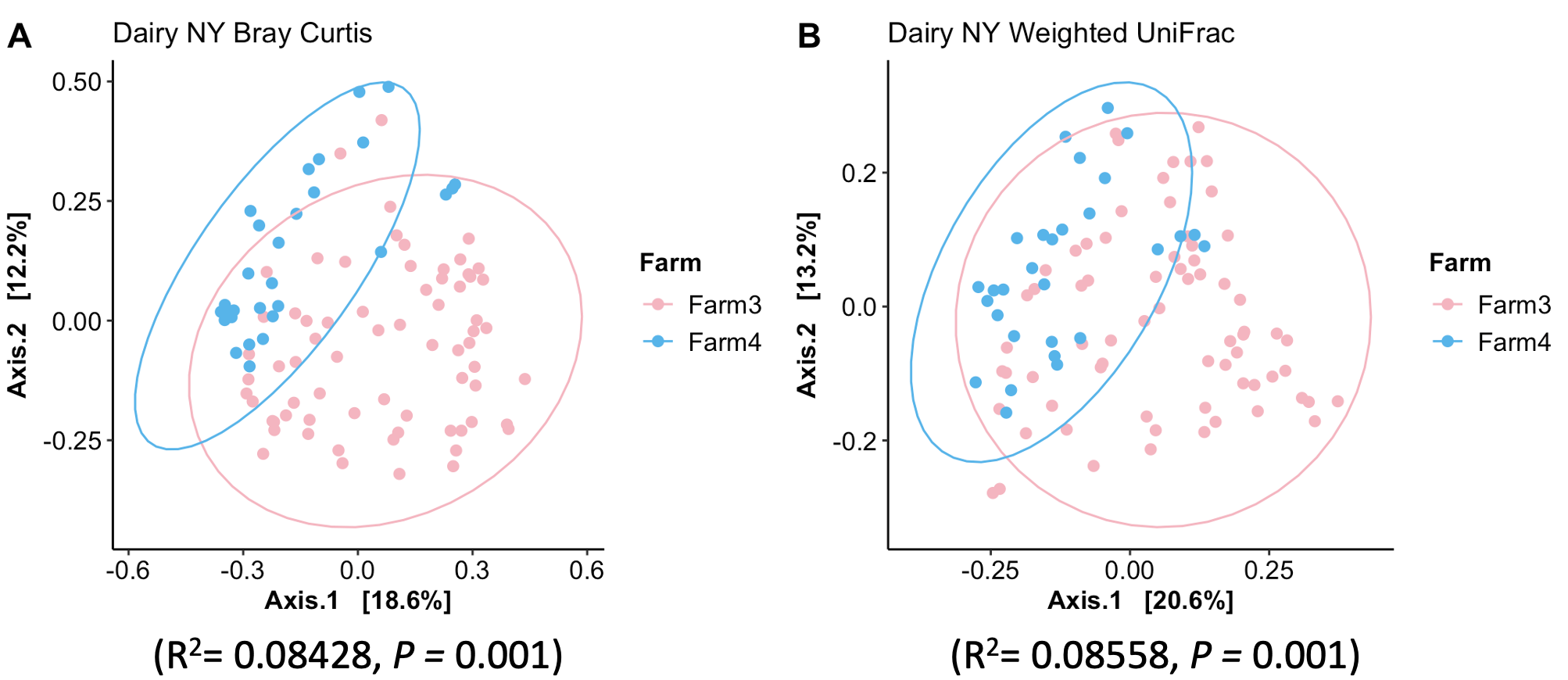


**D**

**C**

**Figure S6.** Beta diversity comparison between all five dairy farms **(a-b)** and specifically the two NY farms using Bray Curtis **(c)** and Weighted UniFrac **(d).**

**Comparison analysis between BRD-pathobiont prevalence and relative abundance between detected via 16S rRNA sequencing and qPCR**

As a comparative analysis, the prevalence and relative abundance of ASVs identified as *Histophilus, P. multocida, M. haemolytica*, and *Mycoplasma* from the 16S rRNA data were compared to the BRD-pathobionts' abundance and prevalence quantified via qPCR. In the 16S rRNA sequencing data, no ASVs were identified as *H. somni* or *M. bovis*; thus, we used classification at the genus level (Figure S7). From the qPCR data, the pathobiont *H. somni* was the most prevalent among all the samples; however, detection of this microbe based on 16S rRNA sequencing was below 50% and completely absent in the CA samples. The prevalence of *M. haemolytica* measured via qPCR and 16S rRNA sequencing was similar in TX and NY samples; nonetheless, detection of this microbe in the IN and CA was low compared to the values identified via qPCR (Figure S7). In the case of *M. bovis*, the prevalence of this microbe was low among all farms (<25%), but when targeting the prevalence of *Mycoplasma*, the presence/absence of this microbe increased. Specifically, samples from TX had a higher prevalence of *Mycoplasma* (>50%), whereas the other farms had a prevalence of <25%. Lastly, the prevalence of *P. multocida* identified via 16S rRNA and qPCR was similar for both groups and depending on the farm (Figure S4.6). In addition to the prevalence test, a correlation analysis was conducted to examine the association between the relative abundance of each pathobiont measured by 16S rRNA sequencing and the relative abundance based on the total bacteria count per sample (16S rRNA gene copy number). This examination aimed to assess if the relative abundance detected by the qPCR assay (which has higher sensitivity than 16S rRNA sequencing) aligns with the relative abundance identified by 16S rRNA sequencing. No significant association was found for *M. bovis*. For *P. multocida, M. haemolytica*, and *H. somni*, there were notable positive associations identified between the relative abundance predicted by 16S rRNA sequencing and the relative abundance of each microbe based on the total bacteria count quantified using qPCR (16S rRNA qPCR assay) (Figure S8). These associations suggest that a higher relative abundance of each microbe predicted by 16S rRNA sequencing is similarly detected by the qPCR assays. Moreover, these significant associations were consistently present when the samples were separated based on disease status (Figure S8). However, the strengths of these associations are relatively low: 0.65 for both *P. multocida*, 0.39 for *M. haemolytica* in the apparently healthy and BRD-affected groups, and the association effect of *H. somni* in the apparently healthy group was 0.44, while in the BRD-affected group it was 0.48.


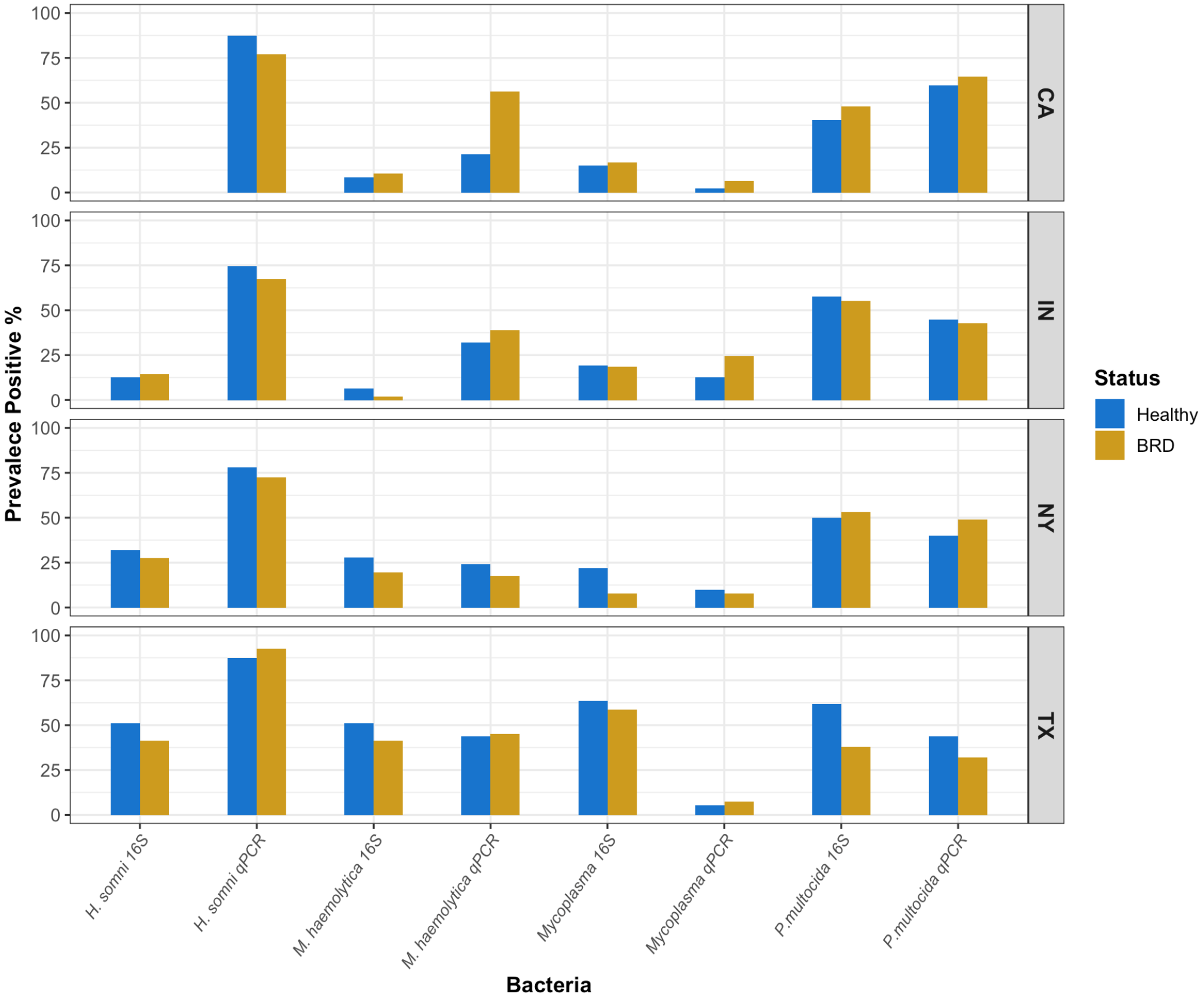


**Figure S7.** Prevalence of the BRD-pathobionts identified via 16S rRNA sequencing and quantified via qPCR based on disease status and farm.


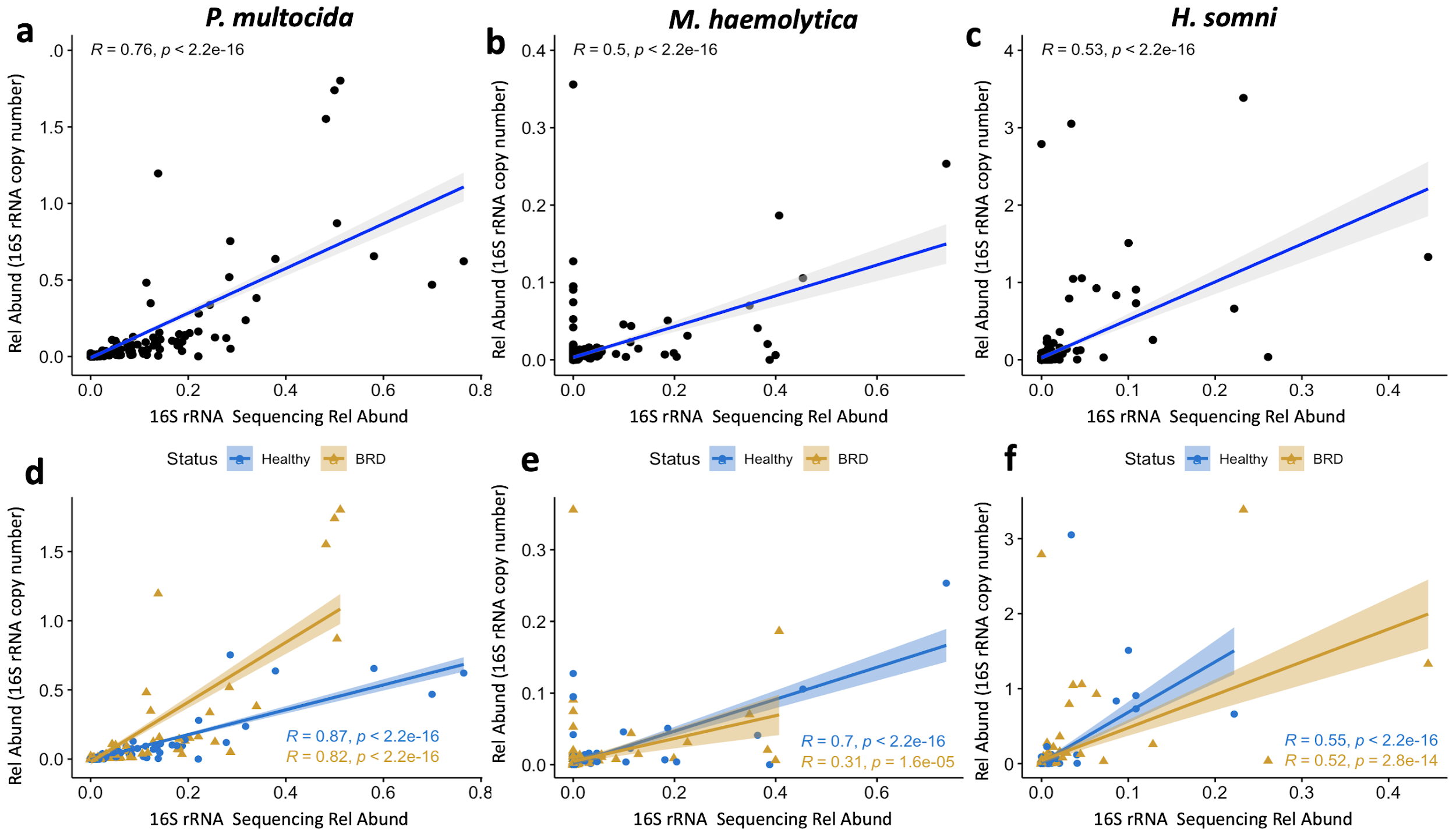


**Figure S8.** Correlation between the BDR-pathobionts relative abundance predicted by 16S rRNA and the relative abundance based on 16S rRNA copy number (log_10_) **(a-c)**. Correlation between the BDR-pathobionts relative abundance predicted by 16S rRNA and the relative abundance based on 16S rRNA copy number (log_10_) for each disease status **(d-f).**
